# Supplementary material for: Abstracts from the Thirteenth Rambam Research Day, December 22, 2016
Source: Rambam Maimonides Med J. 2017 Jan 30;8(1):e0014. doi: 10.5041/RMMJ.10289 (PMC5298375; doi:10.5041/RMMJ.10289)
Supplement: Related texts

# Abstract: About Rambam Research Day

**Published:** 2017-01

We are proud to introduce you to the Thirteenth Rambam Research Day, now established as a key annual event at Rambam Health Care Campus (Rambam), reflecting the diverse research activities on our campus.

Integral to Rambam’s research activities are several competitive research funding programs that Rambam administers: The “Ofakim” program, which provides start-up and bridge research funding for young staff physicians with strong research credentials and plans, and the “Atidim” program, which is directed towards research training support for our residents, or, as we call them in Israel, “Mitmachim.” The Atidim program continues to receive wide recognition and serves as an especially important example of Rambam’s dedication to medical trainees and the development of physician scientists.

Two new competitive research grants were recently initiated. In 2015 the “MAOF” program for Rambam’s nursing staff was initiated. Grants of up to NIS 10,000 are awarded to support research and innovative projects of the nursing staff. “MALKAM,” initiated in 2016, offers up to NIS 20,000 for clinical research projects performed by residents. Both of these programs will stir up additional research initiatives from new sectors of the hospital staff and further contribute to the advancement of medical practice at Rambam.

Combined, these research program are critical components of personal professional development for Rambam’s medical personnel.

We would like to acknowledge the excellent collaboration and the fruitful research ties between Rambam Health Care Campus and the Technion–Israel Institute of Technology, in particular The Ruth & Bruce Rappaport Faculty of Medicine. We would also like to thank Professor Chaim (Howard) Cedar from the Hebrew University of Jerusalem as the Chair of our Scientific Advisory Board.

We are proud of the scientific abstracts that were presented at the meeting, and which compose this Supplement to *Rambam Maimonides Medical Journal*. The abstracts cover all areas of medicine. They reflect the rich spectrum of clinical, applied, and basic research endeavors that characterize our medical center’s activities. Indeed, our primary mission of bringing health care to the community is achieved as we combine these research discoveries with excellence in teaching.

We would also like to recognize the dedicated staff of Rambam’s Research Division, including the Institutional Review Board (IRB), and the Office for Promotion of Research; together they are responsible for the coordination and oversight of all research activities at Rambam, including clinical research, applied research, competitive grants management, patent protection, institutional review board ethical approvals, and bringing forward innovative ideas to clinical practice. We value collaboration with industry, which serves as an integral part of health care progress; over the years, several Rambam-originated research projects have led to remarkable success in Israel, with practical application to health care.

We are particularly pleased to see this Supplement to *Rambam Maimonides Medical Journal*, which has now become a tradition at the *Rambam Maimonides Medical Journal*, for the awareness and promotion of research activities in Israel, particularly at Rambam. Supporting Rambam Research Day via *Rambam Maimonides Medical Journal* is another way we show commitment to our patients, via the furthering of health care facilitated by progress in science, innovation, and technology.

**Rafi Beyar, M.D., D.Sc.** *Director, Rambam Health Care Campus and Associate Editor, Rambam Maimonides Medical Journal***Yehuda Chowers, M.D.** *Director, Clinical Research Institute at Rambam, Rambam Health Care Campus***Karl Skorecki, M.D.** *Director, Medical & Research Development, Rambam Health Care Campus and Associate Editor, Rambam Maimonides Medical Journal***Ehud Klein, M.D.** *Director, Research Division, Rambam Health Care Campus*

# Abstract: Rambam Health Care Campus Research Day Organizing Committee

**Published:** 2017-01

**Professor Ehud Klein**

Director, Research Division

**Professor Shraga Blazer**

Director, Department of Neonatology, and Editor-in-Chief, Rambam Maimonides Medical Journal

**Professor Benjamin Brenner**

Director, Department of Hematology and Bone Marrow Transplantation

**Professor Myriam Weyl Ben-Arush**

Director, Pediatric Division, Ruth Rappaport Children’s Hospital

Director, Pediatric Hematology and Oncology

**Professor Shimon Meretyk**

Chair, Division of Surgery

**Professor Zeev Weiner**

Director, Department of Obstetrics and Gynecology

**Professor Eldad Dann**

Director, Transfusion Service & Aphaeresis

**Dr Yael Shahor**

Deputy Director, Pediatric Division, Ruth Rappaport Children’s Hospital

Attending Physician, Infectious Disease

**Assistant Professor Ron Beloosesky**

Director, Prenatal Ultrasound Unit, Department of Obstetrics and Gynecology

**Clinical Assistant Professor Tony Karram**

Director, Endovascular Surgery and Vascular Access Unit, Division of Surgery

# Abstract: Program for the Thirteenth Rambam Research Day

**Published:** 2017-01

**Thursday, December 22, 2016**

Rambam Health Care Campus, Haifa, Israel

07.00 **Meet & Greet**

07.30 **Opening Session: Greetings**

**Professor Rafi Beyar**

Director, Rambam Health Care Campus

**Professor Eliezer Shalev**

Dean, The Ruth & Bruce Rappaport Faculty of Medicine, Technion–Israel Institute of Technology (IIT), Haifa, Israel

**Professor Karl Skorecki**

Director of Medical and Research Development, Rambam Health Care Campus

**Professor Yehuda Chowers**

Director, The Clinical Research Institute at Rambam (CRIR), at Rambam Health Care Campus

**Professor Ehud Klein**

Director, Research Division, Rambam Health Care Campus

**Honorary Lecturer**

07.50 **Professor Alon Chen**

Chair, Department of Neurobiology, Weizmann Institute of Science, Rehovot, Israel

Director, Department of Stress Neurobiology and Neurogenetics, Max-Planck Institute of Psychiatry, Munich, Germany

**Response to Stress, at the Crossroad of Gene–Environment Interaction**

**Awardsfor Rambam Research Day Oral Presentations Plenary Lectures**

08.45 **Derek LeRoith, M.D., Ph.D.**

Diabetes and Metabolism Clinical Research Center of Excellence, Clinical Research Institute at Rambam (CRIR)

08.55 **Ron Beloosesky, M.D.**

Department of Obstetrics and Gynecology

09.05 **Zvi Peled, M.D.**

Department of Cardiac Surgery

09.15 **Khetam Hussein, M.D.**

Infectious Disease Institute

09.25 **Michal Gur, M.D.**

Pediatric Pulmonary Institute and CF Center

09.40 **Barak Levit, M.D.**

Department of Surgery

09.50 **Carmit Steinberg, M.A.**

Pediatric Emergency Department

10.00 **Tareq Aro, M.D.**

Department of Urology

10.15 **Break**

10.45 **Jacob T. Cohen, M.D.**

Department of Otolaryngology Head and Neck Surgery

10.55 **Michal Hayun, Ph.D.**

Department of Hematology and Bone Marrow Transplantation

11.05 **Amnon Amit, M.D.**

Department of Obstetrics and Gynecology

11.15 **Adi Shapira, M.D.**

Department of Pediatric Hematology-Oncology

11.30 **Sara Selig, Ph.D.**

Molecular Medicine Laboratory

11.40 **Ofer Fainaru, M.D., Ph.D.**

Department of Obstetrics and Gynecology

11.50 **Oren Caspi, M.D., Ph.D.**

Cardiology Department

12.00 **Tova Hershkovitz, M.D.**

The Genetic Institute

12.10 **Closing Session**

**Concluding Remarks Announcement of**

Rambam Atidim 2016 Recipients
Rambam Ofakim 2016 Recipients
Rambam MAOF 2016 Recipients
Rambam MALKAM 2016 Recipients

**Announcement of**

Rambam Innovation Fund (Nitzoz) 2016 Awardees

# Abstract: Response to Stress, at the Crossroad of Gene–Environment Interaction

**Published:** 2017-01

**Authors:** Alon Chen

**Affiliations:** Chair, Department of Neurobiology, Weizmann Institute of Science, Rehovot, Israel; and Director, Department of Stress Neurobiology and Neurogenetics, Max-Planck Institute of Psychiatry, Munich, Germany

The biological response to stress is concerned with the maintenance of homeostasis in the presence of real or perceived challenges. This process requires numerous adaptive responses, involving changes in the central nervous and neuroendocrine systems. When a situation is perceived as stressful, the brain activates many neuronal circuits, linking centers involved in sensory, motor, autonomic, neuroendocrine, cognitive, and emotional functions in order to adapt to the demand. However, the details of the pathways by which the brain translates stressful stimuli into the final, integrated biological response are not completely understood. Nevertheless, it is clear that dysregulation of these physiological responses to stress can have severe psychological and physiological consequences, and there is substantial evidence to suggest that inappropriate regulation, disproportional intensity, or chronic and/or irreversible activation of the stress response is linked to the etiology and pathophysiology of anxiety, depression, and metabolic-related disorders. Understanding the neurobiology of stress by focusing on the specific genes and brain circuits, which are associated with, or altered by, the stress response, will provide important insights into the brain mechanisms by which stress affects psychological and physiological disorders. The long-term goal of our research is to elucidate the pathways by which stress is perceived, processed, and transduced into neuroendocrine and behavioral responses. We are using integrated molecular (genetics and epigenetics), biochemical, physiological, and behavioral methods, with focus on the generation of mutant mouse models as an *in vivo* tool, to study the roles of specific stress-linked genes and brain circuits in coordinating the neuroendocrine, autonomic, and behavioral responses to stressful challenges.

# Abstract: Evaluation of Microscopic Changes in Fallopian Tubes of BRCA Mutation Carriers by Morphometric Analysis of Histological Slides

**Published:** 2017-01

**Authors:** Amnon Amit, Edmond Sabo, Yaniv Zohar, Einat Trugman, Irena Pranovich, Ari Reiss, Geula Klorin

**Affiliations:** 1 Department of Obstetrics and Gynecology, Rambam Medical Center, Haifa, Israel; 2 Institute of Pathology, Rambam Medical Center, Haifa, Israel; 3 Ruth & Bruce Rappaport Faculty of Medicine, Technion–Israel Institute of Technology, Haifa, Israel

## Introduction

Hereditary ovarian cancer is, in part, related to mutations in BRCA genes; currently, there is no method for early diagnosis. Some of the serous ovarian tumors are hypothesized to stem from fallopian tube fimbria cells. Using a novel method of computerized morphometry of the fimbrial epithelium, this study aims to detect morphologic differences in non-cancerous fimbriae between BRCA-mutation carriers and non-carriers, and between healthy and serous ovarian cancer patients.

## Materials and Methods

24 fimbriae from healthy women (13 BRCA-pos, 11 BRCA-neg) and 21 fimbriae from women with serous ovarian cancer (10 BRCA-pos, 11 BRCA-neg) reported as “normal” by H&E examination were subjected to computerized histomorphometric analysis. A fast Fourier transformation (FFT) was applied to images of fimbrial epithelium, and the FFT two-dimensional frequency maps were subsequently quantified for nuclear orientation and planar distribution by a co-occurrence matrix analysis. Additional analysis of nuclear contour was applied on the fimbriae of the healthy women.

## Results

Significant differences were found between the BRCA carriers and non-carriers in the healthy women group. In the ovarian cancer group, no significant differences were found between BRCA carriers and non-carriers. Lastly, significant differences were detected between the healthy and ovarian cancer groups, regardless of the BRCA mutational status.

## Conclusions

Using this novel method, which combined FFT with co-occurrence matrix analysis, we were able to demonstrate differences in morphometric characteristics in the fimbriae between healthy and ovarian cancer patients, and between BRCA mutation carriers and non-carriers. The clinical significance of these observations should be further studied.

# Abstract: Comparing the Burden and Suffering from Primary Percutaneous Nephrostomy Tubes and Ureteral Stents

**Published:** 2017-01

**Authors:** Tareq Aro, Michael Mullerad, Shadi Badaan, Alexander Kastin, Ariel Zisman, Akram Assadi, Oleg Goldin, Gilad E. Amiel

**Affiliations:** Department of Urology, Rambam Medical Center, Haifa, Israel

## Introduction

Drainage of an obstructed kidney can be performed percutaneously using a percutaneous nephrostomy (PCN) or endoscopically via a ureteral stent. Each medical center has their preferences, and sometimes a patient’s choice dictates the type of drainage.

## Objective

We aimed to compare both types of drainage in terms of complications, number of emergency department (ED) visits, readmissions, and burden to the patients.

## Materials and Methods

A retrospective analysis of a matched cohort of 100 patients who underwent an insertion of PCN compared to 100 patients who received a ureteral stent. Patients with risk factors for complications were excluded. Comparisons were made between types of complications and number of ED visits and hospitalizations.

Statistical significance was determined for categorical data using Fisher’s exact test with a two-tailed *P* value <0.05.

## Results

In both groups, 66% were males and the median age was 51 years. The average drainage time was 84 days with PCN and 85 days with a stent (median 75.5 and 80.5 days, respectively). In patients who visited the ED, the main complications with PCN were urinary tract infection (UTI) (36%, *n*=27) and obstruction (23%, *n*=22), whereas in patients with stents they were pain (57.5%, *n*=23) and UTI (32.5%, *n*=13). The median time to the first complication from the time of drainage in the PCN group was 27 days compared to 20 days in the stent group.

Patients treated with ureteral stents had significantly fewer visits to the ED and admissions to the hospital (Table 1).

**Table 1:** Aro et al. Emergency Department Visits and Hospitalization for Both Groups.

	Visited the ED	Hospitalized at Least Once	More than One ED Visit*	Overall ED Visits	Overal Hospitalizations
PCN (*n*=100)	44%	29%	43%	75	33
Ureteral stent (*n*=100)	29%	13%	34%	40	15
*P* value	0.039	0.0087			

* of patients who had visited the ED at least once.

ED, emergency department; PCN, percutaneous nephrostomy.

Based on the data (Table 1), for every 100 patients drained with a PCN for one month we can expect 27 visits to the ED in comparison to 14 visits by patients drained with a stent, and 12 PCN patients will be admitted compared to 5 patients with stents.

## Conclusions

In our retrospective comparison, we found that patients drained with a ureteral stent for the same period of time as PCN had fewer complications, fewer visits to the ED, and fewer hospitalizations.

# Abstract: Maternal Inflammation-Induced Offspring Cerebral Injury Is Prevented by Magnesium Sulfate

**Published:** 2017-01

**Authors:** Ron Beloosesky, Nizar Khatib, Yuval Ginsberg, Zeev Weiner

**Affiliations:** Department of Obstetrics and Gynecology, Rambam Medical Center, Haifa, Israel

## Introduction

Fetal and/or maternal infectious processes may be associated with newborn neurologic injury. Recent data suggest that Mg may protect the preterm fetus from cerebral palsy. As infection/inflammation may be etiologic in preterm labor, we sought to assess the inflammation-associated neuroprotective potential of Mg. We examined the effect of Mg on prevention of maternal lipopolysaccharide (LPS)-induced neonatal brain injury using MRI.

## Materials and Methods

Pregnant Sprague-Dawley rats at 18 days’ gestation received i.p. LPS (500 μg/kg) or saline at time 0. Dams were randomized to treatment with s.c. saline or magnesium sulfate (270 mg/kg loading followed by 27 mg/kg every 20 min) for 2 hours prior to and following the i.p. LPS or saline. Pups were delivered spontaneously (e21) and allowed to mature until postnatal day 25. Female offspring (4–8 per group) were examined under isoflurane anesthesia by MRI brain imaging and analyzed using voxel-based analysis (VBA) after spatial normalization. T2 relaxation time was used to assess for white matter injury and diffusion tensor imaging for fractional anisotropy (FA) comparison.

## Results

Offspring of LPS-treated dams exhibited (1) significantly increased T2 levels, and (2) reduced FA levels in white and gray (e.g. corpus callosum, thalamus, hippocampus) matter, consistent with diffuse cerebral injury. In contrast, offspring of Mg-treated LPS dams demonstrated similar T2 and FA levels as controls in both white and gray matter.

## Conclusions

Treatment with Mg significantly reduced evidence of neonatal brain injury associated with maternal LPS. These studies suggest that maternal Mg therapy may be most effective in human preterm deliveries associated with maternal/fetal inflammation.

# Abstract: Generation of Induced Pluripotent Stem Cell-Based *In Vivo* Models for Studying Cardiomyopathies

**Published:** 2017-01

**Authors:** Oren Caspi, Snizhana Chrona, Inbar Budniatzky, Irit Huber, Amit Gruber, Amira Gepstein, Gil Arbel, Lior Gepstein

**Affiliations:** 1 Sohnis Family Research Laboratory for Cardiac Electrophysiology and Regenerative Medicine, The Ruth & Bruce Rappaport Faculty of Medicine, Technion–Israel Institute of Technology, Haifa, Israel; 2 Department of Cardiology, Rambam Medical Center, Haifa, Israel

## Introduction

The study of cardiomyopathies is hampered both by the inability to assess the myocardial tissue early during disease development and the lack of suitable models. The advent of human induced pluripotent stem cell (hiPSC) technology may provide essential means for overcoming this hurdle, since it allows deriving and studying human cardiomyocytes that carry the disease-causing phenotype. However, hiPSCs based *in vitro,* “disease in dish models”, are limited by the relative structural, functional, and metabolic immaturity of the cardiomyocytes, the inability to assess for the effects of systemically delivered therapeutic interventions, and the lack of supporting microenvironment.

## Objective

In the current study, we aimed to establish hiPSC-based *in vivo* models for cardiomyopathies to assess the ability of such a strategy to (1) unmask the phenotype of adult-onset cardiomyopathies (e.g. arrhythmogenic right ventricular cardiomyopathy); (2) obtain functional information from the engrafted hiPSC-derived cardiomyocytes (hiPSC-CMs), and; (3) assess the effect of systemically delivered therapies on hiPSC-CMs.

## Materials and Methods

Heterotopic and orthotopic strategies were used to transplant hiPSC-derived cardiomyocytes (following 15–30 days of differentiation) to NOD-SCID mice. Orthotopic experiments were conducted by direct myocardial delivery of the hiPSC-CMs. For heterotopic experiments hiPSC-CMs were transduced with genetically encoded calcium indicator, GCAMP6, and were transplanted into the transparent dermal layer of the outer ear of NOD-SCID mice.

## Results

Orthotopic transplantation of arrhythmogenic right ventricular cardiomyopathy (ARVC) hiPSC-CMs to the mouse myocardium resulted in stable and discrete grafted tissue islands. Phenotypic characterization using confocal immunofluorescence demonstrated that transplanted ARVC-hiPSC-CMs recapitulated the disease phenotype within the mouse myocardium when compared to control cells. Heterotopic transplantation demonstrated that transplanted cells survived within the dermal thin layer of the outer ear (for up to 3 months) and their fluorescent signal was detectable in live mice using an optical mapping system.

## Conclusions

Cardiomyopathy modeling based on hiPSCs may be conducted not only in the *in vitro* environment but also using heterotopic and orthotopic strategies for *in vivo* modeling. Based on these strategies, structural and functional alterations associated with the cardiomyopathies may be recapitulated, and the effect of therapeutic interventions can be assessed in a clinically relevant manner.

# Abstract: Cold Atmospheric Plasma for Cancer Therapy

**Published:** 2017-01

**Authors:** Jacob T. Cohen, Yoav Binenbaum, Gil Ziv

**Affiliations:** 1 Department of Otolaryngology Head and Neck Surgery, Rambam Medical Center, Haifa, Israel; 2 The Laboratory for Applied Cancer Research, Rambam Medical Center, Haifa, Israel; 3 The Ruth & Bruce Rappaport Faculty of Medicine, Technion–Israel Institute of Technology, Haifa, Israel

## Introduction

Plasma is ionized gas that is typically generated in high-temperature laboratory conditions. Recent progress in plasma technology has led to the creation of cold atmospheric plasma (CAP), with an ion temperature close to room temperature. CAP has tremendous applications in biomedical engineering and can potentially offer surgical options that allow specific cell removal without influencing the whole tissue. We developed a hand-held device for inner body delivery of cold plasma.

## Objective

To evaluate the efficacy of our CAP design on cancer, *in vitro* and *in vivo*.

## Materials and Methods

Our team has been developing and using CAP devices *in vitro* and *in vivo*. Minimal CAP treatment time for killing 50% of cells was evaluated for different cell lines *in vitro*. Reactive oxygen species (ROS)-induced DNA damage was assessed. *In vivo* CAP treatments on mice carrying melanoma, adenocarcinoma, and squamous cell carcinoma were evaluated.

## Results

*In vitro*, 60 seconds of CAP application resulted in a 50% reduction in cancer cell population within 48–72 hours of treatment. Our preliminary data suggest that enzymes involved in reactive oxygen species scavenging are up-regulated in CAP-treated cells, most probably indicating a ROS-mediated apoptosis mechanism. Malignant melanoma flank tumors treated by CAP have decreased in size by 40%–50%, while adjacent healthy skin tissue was unaffected by the treatment. In colon adenocarcinoma flank tumors, 8 minutes of treatment could eradicate tumors completely. Same results were noticed in head and neck squamous cell cancer.

## Conclusions

CAP treatment demonstrates a selective effect, causing apoptosis primarily in cancer cells. It is envisioned that adaptation of CAP technology for clinical use may provide a new modality for the treatment of solid tumors.

# Abstract: Repair of Genetic Diseases at the Level of Preimplantation Embryos

**Published:** 2017-01

**Authors:** Ofer Fainaru, Rivi Hertz, Shahar Kol

**Affiliations:** IVF unit and Laboratory for Reproductive Sciences, Clinical Research Institute at Rambam (CRIR), Rambam Medical Center, Haifa, Israel

## Introduction

Genetic diseases pose an immense burden to inflicted individuals. Currently, only preventive therapy to identify carriers and healthy embryos using pre-gestational diagnosis is being practiced; often normal embryos or a viable pregnancy cannot be achieved. Therefore, gene editing holds promise to become the only solution for selected carrier couples. Using the CRISPR/Cas9 system that has revolutionized the field in recent years, it is now possible to target gene-editing tools to a specific mutated gene, snipping it out, then replacing the gene with its normal counterpart. We have recently performed proof-of-concept gene-editing experiments using CRISPR/Cas9 in mouse zygotes. Direct co-injection of RNA encoding the Cas9 protein and locus-specific guide RNAs (sgRNAs) into zygotes allowed for efficient generation of mice carrying mutations in the tyrosinase gene causing mouse albinism.

## Materials and Methods

Two sgRNA plasmids targeting different regions of the tyrosinase gene were designed (Try-E1-S2-B and Try-E1-S10-A). Mouse zygotes were derived from C57BL/6 donor mice that were superovulated and mated with C57BL/6 or albino (C57BL/6J-Tyrc-2J) males. The two sgRNAs and the Cas9 mRNA were co-injected into the cytoplasm and nucleus of 200 mouse zygotes. The zygotes were transferred into pseudopregnant foster mothers’ oviducts (30 per mouse). Functional mutants, with perturbation of the tyrosinase gene, should have pink rather than black eyes. Since the mutation is recessive, embryos derived from wild-type (B6) males or albino (C57BL/6J-Tyrc-2J) males allowed for detection of homozygous as well as heterozygous mutations.

## Results

In E13.5 embryos (B6xB6) injected with a single guide to exon 1 of the Tyr gene, we detected pink-eyed pups. These pups are a result of *homozygous* mutations, i.e. indels in the Tyr gene on both alleles. We also detected E15.5 embryos (B6-TyrCxB6) injected with two guide RNAs to the Tyr gene. The pink-eyed pups are a result of *heterozygous* interruption of the Tyr gene: either an indel or a deletion of part of exon 1 and 2 on one or both alleles**.**

## Conclusions

The above experiments demonstrate our capability specifically to edit single genes at the level of the preimplantation embryo. We thus propose to implement this exciting new technology in human embryos for the future treatment of human genetic diseases.

# Abstract: Lung Clearance Index (LCI) in Patients with Bronchiolitis Obliterans: A Preliminary Report and Comparison to Cystic Fibrosis Patients

**Published:** 2017-01

**Authors:** Michal Gur, Karin Yaacoby-Bianu, Anat Ilivitzki, Ronen Bar–Yoseph, Vered Nir, Fahed Hakim, Yazeed Toukan, Lea Bentur

**Affiliations:** 1 Pediatric Pulmonary Institute and CF Center, Ruth Rappaport Children’s Hospital, Rambam Medical Center, Haifa, Israel; 2 Pediatric Radiology Unit, Ruth Rappaport Children’s Hospital, Rambam Medical Center, Haifa, Israel; 3 The Ruth & Bruce Rappaport Faculty of Medicine Technion–Israel Institute of Technology, Haifa, Israel

## Introduction

Bronchiolitis obliterans (BO) is a chronic airway disease following an insult to the lower respiratory tract. Lung clearance index (LCI) measures ventilation inhomogeneity and has been studied in cystic fibrosis (CF).

## Objectives

We aimed to evaluate LCI in BO and to compare it to LCI in CF patients.

## Materials and Methods

LCI was measured in BO patients, compared to CF patients, and correlated with spirometry and CT findings.

## Results

Twenty BO patients and 26 CF patients (with similar mean age and BMI) underwent evaluation. FEV1% predicted and FEF 25%–75% predicted were significantly lower in the BO group (60.5±17.8 vs. 72.7±20.7 , *P*=0.041 and 42.8±22.8 vs. 66.4±37.4 , *P*=0.017, respectively). In both groups, LCI was inversely correlated with FVC, FEV1, and FEF 25–75 % predicted. LCI % predicted was slightly higher (190.4±63.5 vs. 164.9±39.4, *P*=0.1) and FRC % predicted gas (FRC measured by multiple breath washout) was significantly higher in the BO group (92.5±35.9 vs. 71.3±18 , *P*=0.014). The statistical association between the lower FEF 25%–75% values and the higher LCI values was stronger in BO patients.

## Conclusions

Similar to CF, LCI may provide estimation of ventilation inhomogeneity in BO. The results indicate greater small airway involvement and air trapping in BO. Further prospective longitudinal studies evaluating the correlation of LCI measurements with multiple clinical and physiological parameters, including whole-body plethysmography, should be performed to assess the clinical benefit of LCI measurement in BO.

# Abstract: Clonal Selection in Acute Myeloid Leukemia Correlates with Differentiation Capacity and Sensitivity to Chemotherapy

**Published:** 2017-01

**Authors:** Michal Hayun, Maria Zaatra, Dvora Sahar, Dina Rosenberg, Igal Louria-Hayon, Yishai Ofran

**Affiliations:** 1 Hematology Research Center, Rambam Medical Center, Haifa, Israel; 2 Ruth & Bruce Rappaport Faculty of Medicine, Technion–Israel Institute of Technology, Haifa, Israel; 3 Hematology Laboratory, Rambam Medical Center, Haifa, Israel; 4 Department of Hematology and Bone Marrow Transplantation, Rambam Medical Center, Haifa, Israel

## Introduction

In acute myeloid leukemia (AML) patients, leukemic sub-clones which survive chemotherapy may lead to relapse. No mechanism linking clonal selection to a specific leukemia-associated genetic aberration is known. We hypothesized that clonal selection might result from differences in differentiation capacity amongst sub-clones and that chemo-resistance is not necessarily directly affected by specific mutations.

## Materials and Methods

Kasumi-1 leukemia cell line was sorted by FACSAria to CD34+CD117+ and CD34-CD117+ sub-populations and exposed to escalating doses of daunorubicin (DNR) and cytarabine (ARA-C). Cell viability was determined by alamarBlue assay; apoptosis was assessed by annexin-V and propidium iodide (PI) staining. Targeted gene sequencing was conducted using Ion Torrent™ PGM System. Allelic frequency of mutations in CD34+ and CD34- sub-populations was measured. FLT3-ITD was sequenced using the GeneScan method.

## Results

Following sorting, cultured kasumi-1 CD34+ cells partly differentiated into CD34− cells, while the CD34− sub-population maintained its phenotype, indicating that the CD34− subpopulation is more maturated. Only the CD34+ sub-population was resistant to chemotherapy: the CD34− sub-population exhibited a 4-fold higher apoptosis rate than CD34+ after exposure to ARA-C. Daunorubicin resulted in a 2.5-fold higher apoptosis in the CD34− compared to the CD34+ sub-population.

Bone-marrow samples from diagnosis and relapse of three AML patients were sorted to CD34+CD117+ and CD34-CD117+ blast subpopulations, and their sub-clonal composition was compared. At diagnosis, most of the blasts had CD34− phenotype, while at relapse the CD34+ population grew. The FLT3-ITD allelic ratio (AR) was higher in CD34+ cells. In the first patient, FLT3-ITD was not detected at diagnosis, while at relapse FLT3-ITD was solely presented in the CD34+ sub-population. In the second and the third patient, the dominant clone at diagnosis was NPM1mutFLT3wtDNMT3Awt and >95% cells were CD34−. At relapse, the FLT3-ITD DNMT3Awt clone became dominant and was mostly located within the CD34+ population. Another sub-clone, FLT3-ITD DNMT3Amut, resided only within the CD34+ population. In addition, in 4 out of 8 FLT3-ITD patients, the carrying FLT3-ITD clones resided only in one sub-population.

## Conclusions

Chemo-resistance and survival of a specific AML sub-clone correlate with its phenotype and differentiation level. Even genetic aberrations which have no direct interaction with chemotherapy may give rise to clonal selection. Sub-clonal differentiation capacity may affect its sensitivity to chemotherapy.

# Abstract: Loss of Glycine Transporter 1 Causes a Subtype of Glycine Encephalopathy with Arthrogryposis and Mildly Elevated Cerebrospinal Fluid Glycine

**Published:** 2017-01

**Authors:** Tova Hershkovitz, Alina Kurolap, Anja Armbruster, Katharina Hauf, Adi Mory, Tamar Paperna, Ewald Hannappel, Galit Tal, Yusif Nijem, Ella Sella, Muhammad Mahajnah, Anat Ilivitzki, Dov Hershkovitz, Nina Ekhilevitch, Hanna Mandel, Volker Eulenburg, Hagit N. Baris

**Affiliations:** 1 The Genetics Institute, Rambam Medical Center, Haifa, Israel; 2 The Ruth and Bruce Rappaport Faculty of Medicine, Technion–Israel Institute of Technology, Haifa, Israel; 3 Institute of Biochemistry, University of Erlangen-Nuremberg, Erlangen, Germany; 4 Metabolic Unit, Rambam Medical Center, Haifa, Israel; 5 Pediatric and Neonatal Unit, Nazareth Hospital EMMS, Nazareth, Israel; 6 Child Neurology and Development Center, Hillel Yaffe Medical Center, Hadera, Israel; 7 Pediatric Radiology Unit, Radiology Department, Rambam Medical Center, Haifa, Israel; 8 Department of Pathology, Rambam Medical Center, Haifa, Israel

## Background

Glycine serves as a major neurotransmitter by activating inhibitory glycine receptors, and as a co-agonist for excitatory glutamatergic NMDA receptors. Extracellular glycine levels in the central nervous system are regulated by two transporters, GLYT1 and GLYT2. Dysregulation of glycine homeostasis is known to cause hyperekplexia and nonketotic hyperglycinemia. We report two unrelated consanguineous families with children presenting at birth with facial dysmorphism, encephalopathy, arthrogryposis, hypotonia progressing to hypertonicity with startle-like clonus, and respiratory failure. Two probands had mildly elevated cerebrospinal fluid (CSF) glycine with normal serum glycine.

## Materials and Methods

Whole exome analysis was performed on the proband from Family 1. The candidate gene was Sanger sequenced in the proband of Family 2. CSF and blood were analyzed for glycine concentrations by high-pressure liquid chromatography in naive, ALX5407-treated (GlyT1 inhibitor) and GlyT1fl/fl/GFAP-Cre mice.

## Results

We identified loss-of-function mutations in SLC6A9, encoding GLYT1, in both families. Mildly elevated CSF glycine concentrations were observed in ALX5407-treated and GlyT1fl/fl/GFAP-Cre mice (*P*<0.01). Total blood glycine levels were similar in all groups.

## Discussion

We delineate a novel neurogenetic syndrome hallmarked by a unique metabolic phenotype. Our studies in mice demonstrate that the mild elevation in CSF glycine, observed both in patients and mice with inactive GlyT1, is caused by transporter dysfunction. Phenotypes of slc6a9-null mice and zebrafish mutants display similarity to affected humans. Discovery of rare genetic phenotypes not only sheds light on gene function, but also offers new hope for patients’ families, opening preconception treatment options and suggesting targets for palliative care for patients.

# Abstract: What Is the Minimal Intervention Bundle to Stop a CRE Outbreak?

**Published:** 2017-01

**Authors:** Khetam Hussein, Galit Rabino, Orna Eluk, Sigal Warman, Shimon Reisner, Larisa Halif, Michal Paul

**Affiliations:** Infectious Disease Institute, Rambam Medical Center, Haifa, Israel

## Introduction

Rambam has been facing an ongoing carbapenem-resistant Enterobacteriaceae (CRE) outbreak since 2006. We evaluated the minimal infection control (IC) intervention bundle required to curtail the epidemic.

## Materials and Methods

This retrospective quasi-experimental study covered data from 2006 to 2016. Data were collected from prospective IC and hospital databases, including: CRE prevalence and incidence, admissions, mean age, hospital-days (HD), occupancy rate, IC interventions applied, number of rectal surveillance swab cultures, carbapenem consumption, and IC and infectious diseases (ID) staffing. Time series analyses were used to compare the years before and after 2012, based on yearly data.

## Results

The CRE acquisitions, carbapenem consumptions, and surveillance swab samples are illustrated in Figure 1 (next page). The rate of CRE acquisitions increased from 2/100,000 HD patients in 2005, to 86.2 and 80.2 in 2008 and 2011, respectively. Since then the rate has fallen to 20.2 new cases in 2016 (P<0.001). The breaking point was observed in 2012. Until then the interventions applied (cohorting of CRE patients and staff, surveillance on admission for patients at risk, and hospital administration involvement) failed to break the ascending curve. Surveillance during hospitalization in high-risk departments, carbapenem restriction, and a hospital-wide hand hygiene campaign including observations and feedback were applied as a bundle since 2012, as necessary steps for limiting the epidemic. Decreasing CRE incidence was observed with an ID/IC staffing of 1.2–1.5/100 beds and 20,000–36,000 yearly surveillance samples.

**Figure 1:** Hussein et al. Carbapenem Consumption Defined Daily Dose (DDD)/1000HD; Surveillance Swabs Absolute Number/1000; CRE Acquisitions/100,000 HD.

## Conclusions

No isolated single intervention can stop a CRE outbreak. A multifaceted hospital-wide intervention program is needed in order to reduce CRE spread in the hospital setting.

# Abstract: CD24 Cell Surface Expression in MVT1 Mammary Cancer Cells Serves as a Biomarker for Sensitivity to Anti-IGF-1R Therapy

**Published:** 2017-01

**Authors:** Ran Rostoker, Sarit Ben-Shmuel, Elina Isakov, Rola Rashed, Zila Shen Orr, Derek LeRoith

**Affiliations:** Diabetes and Metabolism Clinical Research Center of Excellence, Rambam Medical Center, Haifa, Israel; and The Ruth & Bruce Rappaport Faculty of Medicine, Technion–Israel Institute of Technology, Haifa, Israel

## Introduction

The pro-tumorigenic effects of the insulin-like growth factor receptor (IGF-1R) are well described. However, phase 3 clinical trials in unselected patients demonstrated a lack of efficacy for anti-IGF-1R therapy. These findings suggest that predictive biomarkers are warranted in order to identify patients that would benefit from anti-IGF-1R therapeutic strategies.

## Materials and Methods

shRNA vectors were used to determine IGF-1R’s role in mammary tumorigenesis. Control and IGF-1R-knockdown (IGF-1R-KD) cells were sorted using Fluorescence activated cell sorting (FACS) into CD24− and CD24+ subsets and further characterized in vitro. The tumorigenic capacity of each subset was determined following orthotopic inoculation into the mammary fat pad of female mice. Tumor cells were FACS characterized upon sacrifice to determine IGF-1R’s effect on the plasticity of this cell’s phenotype. Metastatic capacity was assessed using the tail vein assay.

## Results

IGF-1R-KD in cancer cells expressing CD24 affect both their morphology (from mesenchymal-like into epithelial-like morphology) and phenotype *in vitro*. Moreover, we demonstrated that IGF-1R-KD abolished CD24+ cells’ capacity to form both mammary tumors and lung metastatic lesions. We found a marked upregulation in CTFG (tumor suppressor) and a significant reduction of SLP1 (tumor promoting) gene expression in the CD24+/IGF-1R-KD cells and tumors. Moreover, we demonstrated that IGF-1R is essential for the maintenance of stem/progenitor-like cancer cells, and we further demonstrated that IGF-1R-KD induces *in vivo* differentiation of the CD24+ cells toward the CD24− phenotype.

## Conclusions

These findings suggest that CD24 cell surface expression may serve as a valuable biomarker to identify mammary tumors that will positively respond to targeted IGF-1R therapies.

# Abstract: Establishing a Definitive Airway in the Trauma Patient by Novice Intubators: A Randomized Crossover Simulation Study

**Published:** 2017-01

**Authors:** Itai Shavit, Barak Levit, Nofar Ben Basat, Dekel Lait, Mostafa Somri, Luis Gaitini

**Affiliations:** 1 Pediatric Emergency Department, Rambam Medical Center (RMC), Haifa, Israel; 2 Ruth & Bruce Rappaport Faculty of Medicine, Technion–Israel Institute of Technology, Haifa, Israel; 3 Department of Surgery, RMC, Haifa, Israel; 4 Department of Anesthesiology, RMC, Haifa, Israel; 5 Anesthesiology Department, Bnai Zion Medical Center, Haifa, Israel

## Introduction

Establishing a definitive airway, defined as a tube placed in the trachea with cuff inflated below the vocal cords, is the standard of care in pre-hospital airway management of the injured patient. However, success rates of intubation with direct laryngoscopy (DL) at the pre-hospital setting are suboptimal, especially in patients with suspected cervical spine injury. We aimed to compare the success rate of tracheal intubation with DL to blind intubation through an intubating-laryngeal mask airway (I-LMA) during manual in-line stabilization of the neck.

## Materials and Methods

A randomized, crossover simulation study was performed. Study participants were 29 military emergency medical technicians undergoing initial training for paramedic status. The primary outcome measure was success rate in one intubation attempt.

## Results

Study subjects had a higher one-attempt success rate with I-LMA than with DL (27/29 vs. 18/29, *P*<0.025, 95% confidence interval (CI) [1.0 to 3.4]), and the I-LMA was assessed as easier to use (*P*<0.0001, 95% CI [0.1 to 2.0]). A significantly longer duration of intubation was found with I-LMA compared to DL (54.2 seconds vs. 42.8 seconds, *P*<0.002, 95% CI [−15.6 to −3.8]). Success rate of I-LMA insertion to the supraglottic space on first attempt was 28/29 (96.5%). Duration of I-LMA insertion was significantly shorter than duration of intubation with DL (26.9 seconds vs. 42.8 seconds, *P*<0.0001, 95% CI [−20.2 to −12.5]).

## Conclusions

Novice intubators instructed in how to perform tracheal intubation during manual in-line stabilization of the neck had higher success rates with the I-LMA than with DL. Study findings suggest that for patients with suspected cervical spine injury, inexperienced providers should use the I-LMA to establish a definitive airway**.**

# Abstract: Respiratory Guard System: New Technology

**Published:** 2017-01

**Authors:** Zvi Peled, Avishai Zisser, Keren Bitton Worms, Victor Kerzman, Oved Cohen, Gil Bolotin

**Affiliations:** 1 Department of Cardiac Surgery, Rambam Medical Center, Haifa, Israel; 2 Department of Anesthesia, Rambam Medical Center, Haifa, Israel

## Introduction

Ventilator-associated pneumonia is a hazardous complication associated with significant morbidity and mortality. The safety and efficacy of the *AnapnoGuard* system, a novel respiratory guard system, as an airway management tool in mechanically ventilated patients was evaluated in this prospective, double-arm, randomized-controlled study.

## Materials and Methods

The study population included 41 patients. Study group patients were connected to the *AnapnoGuard* control unit in which subglottic secretions suction and cuff pressure controls were enabled. In the control group, patients were treated according to the current standard of care. In both groups, the presence of CO2 levels above the cuff was measured by the control unit. The primary end-point of the study was the overall duration and extent of endotracheal tube cuff leakage determined by CO2 area under the curve (receiver operating characteristic (ROC) curve). Secondary end-points included number of cuff pressure measurements within the safely accepted range (24 to 40 cmH2O) and the number of significant CO2 leakage readings.

## Results

The average CO_2_ area under the curve representing CO_2_ leakage calculated for the study group was significantly lower compared to the control group (0.09±0.04 vs. 0.22±0.32, respectively, *P*<0.001). Measurements of significant leakages (CO_2_≥2 mmHg) were significantly lower in the study group than in the control group (0.056 vs. 0.642, respectively, *P*<0.001). Cuff pressure measurements within the safety range in the study group were almost 3 times higher compared to the control group (99.6% vs. 35.1%, *P*<0.001). The average volume of secretions evacuated was 1.8 times higher in the study group compared to the control group (105 mL/day vs. 59 mL/day) (*P*=0.1). No serious adverse events were recorded throughout the study.

## Conclusions

The *AnapnoGuard* system was found to be safe and effective in reducing CO_2_ leaks and maintaining proper cuff pressure. The greater secretion evacuation in the study group may indicate that less secretion leaked down to the bronchial tree.

# Abstract: CRISPR/Cas9-Mediated Correction of DNMT3B in IPSCs Derived from an Immunodeficiency, Centromeric Instability, Facial Anomalies (ICF) Syndrome Patient

**Published:** 2017-01

**Authors:** Shir Toubiana, Miriam Gagliardi, Aya Tzur-Gilat, Hagar Katzir, Adi Tsitiat, Maria R. Matarazzo, Sara Selig

**Affiliations:** 1 Molecular Medicine Laboratory, Rambam Medical Center, Haifa, Israel; 2 The Ruth & Bruce Rappaport Faculty of Medicine, Technion–Israel Institute of Technology, Haifa, Israel; 3 Institute of Genetics and Biophysics, ABT CNR, Naples, Italy

## Introduction

DNA methylation plays important roles in gene expression regulation and heterochromatin assembly. During implantation, DNA methyltransferase 3B (DNMT3B) *de novo* methylates many genomic sites, amongst them repetitive sequences that include subtelomeric regions. Bi-allelic loss-of-function mutations in *DNMT3B* lead to the rare ICF (immunodeficiency, centromeric instability, facial anomalies) syndrome type I, characterized by genome-wide hypomethylation. Due to epigenetic dysregulation of unidentified genes, patients with this syndrome have reduced serum immunoglobulin levels that render them highly susceptible to infections. We have demonstrated previously that ICF cells display hypomethylated subtelomeres, abnormally short telomeres and elevated levels of TERRA, a long non-coding RNA transcribed from these regions. Ectopic expression of wild-type DNMT3B in ICF fibroblasts fails to restore methylation, suggesting that DNMT3B can methylate its targets only at a specific stage during development. Human induced pluripotent stem cells (hiPSCs) mimic the implantation stage and thus are suitable for studying methylation during development. We therefore generated ICF-iPSCs and studied the telomere-specific abnormalities associated with compromised *de novo* methylation. The objective of the current study is to determine whether the methylation defects, both genome-wide and telomere-specific, may be rescued in ICF-iPSCs.

## Materials and Methods

Utilizing the CRIPSR/Cas9 technology, we corrected the mutation in *DNMT3B* in an ICF-iPSC line. Two homozygous repaired clones were obtained.

## Results

Following editing, centromeric repeats were methylated to normal levels, validating the restored activity of DNMT3B. Intriguingly, a subtelomeric region containing the TERRA promoter remained hypomethylated, suggesting a mechanism that protects these regions from *de novo* methylation.

## Conclusions

Further analyses will determine whether the abnormal telomeric phenotype was rescued in the corrected iPSCs. Whole-genome bisulfite sequencing of the original and corrected clones will reveal DNMT3B target sites and indicate whether these subtelomeric regions are uniquely protected from *de novo* methylation in the corrected ICF-iPSCs, and if other genomic regions exhibit a similar fate.

# Abstract: Outcome of Second Allogeneic Hematopoietic Stem Cell Transplant in Children with Malignant and Non-Malignant Underlying Diseases. A Single Center Retrospective Analysis

**Published:** 2017-01

**Authors:** Adi Shapira, Aharon Geffen, Nira Cohen-Arad, Ayelet Ben Barak, Oz Mordechai, Khalil Abdallah, Ronit Elhasid, Iris Porat, Myriam Weyl Ben Arush, Irina Zaidman

**Affiliations:** 1 Department of Pediatric Hematology & Oncology, Ruth Rappaport Children’s Hospital, Haifa, Israel; 2 Department of Pediatric Hematology & Oncology, Dana-Dwek Children’s Hospital, Tel Aviv Sourasky Medical Center, Tel Aviv, Israel

## Introduction

Relapse following hematopoietic stem cell transplant (HSCT) is the leading indication for a second transplant in patients with malignant disease. HSCT has been shown to be superior to chemotherapy alone or palliative measures in these patients. For non-malignant disease a second transplant may be considered for graft failure after a first transplant. Data regarding the outcome of a second HSCT for non-malignant disease are scarce. We retrospectively analyzed 29 patients who underwent a second HSCT, for survival and toxicity data.

## Materials and Methods

Twenty-nine patients (age 0–19 years) who received a second HSCT at our institution during 1998–2015 were included in the analysis. Thirteen patients had an underlying malignancy, and 16 patients were transplanted for non-malignant indications, including inborn errors of metabolism, non-malignant hematologic diseases, and immune deficiency.

## Results

Median follow-up was 14 months (range 1–180). There were 10 deaths (**77**%) in the malignant group: 7 (53%) were due to disease relapse, and 3 (23%) were transplant-related. Fifty percent of deaths occurred within the first year following the second HSCT. In the non-malignant group there were 5 deaths (31%), of which 2 (12%) were attributed to the underlying disease and 3 (18%) were transplant-related. All deaths but one occurred within the first year post HSCT.

## Conclusions

Treatment-related mortality following second HSCT is higher compared to first transplant. The higher survival rate in the nonmalignant group suggests that transplant following graft failure should be considered in patients with otherwise incurable underlying disease. Though the outcome for patients with relapse of malignant disease following HSCT is poor, a second transplant may benefit a subset of these patients. Attempts to achieve complete remission prior to transplant should be made to improve outcome. Due to the small number of patients in our cohort, further multi-center trials are needed.

# Abstract: Involvement of Heparanase in the Pathogenesis of Acute Kidney Injury: Nephroprotective Effects of PG545

**Published:** 2017-01

**Authors:** Zaid Abassi, Shadi Hamoud, Ahmad Hassan, Iyad Khamaysi, Omri Nativ, Samuel Heyman, Rabia Shekh Muhammad, Neta Ilan, Preeti Singh, Israel Vlodavsky, Edward Hammond, Gianluigi Zaza, Antonio Lupo, Maurizio Onisto, Gloria Bellin, Giovani Gambaro, Valentina Masola

**Affiliations:** 1 Department of Physiology, The Ruth & Bruce Rappaport Faculty of Medicine Technion–Israel Institute of Technology (IIT), Haifa, Israel; 2 Department of Laboratory Medicine, Rambam Medical Center, Haifa, Israel; 3 Department of Internal Medicine E, Rambam Medical Center, Haifa, Israel; 4 Department of Internal Medicine A, Rambam Medical Center, Haifa, Israel; 5 Department of Gastroenterology, Rambam Medical Center, Haifa, Israel; 6 Department of Internal Medicine, Hebrew University–Hadassah Medical Center, Jerusalem; 7 Cancer and Vascular Biology Research Center, Ruth & Bruce Rappaport Faculty of Medicine, Technion–IIT, Haifa, Israel; 8 Progen Pharmaceuticals, Brisbane, Queensland, Australia; 9 Renal Unit, Department of Medicine, University Hospital of Verona, Verona, Italy; 10 Department of Biomedical Sciences, University of Padova, Padova, Italy; 11 Columbus-Gemelli Hospital, Catholic University of the Sacred Heart, Roma, Italy

## Introduction

Despite the high prevalence of acute kidney injury (AKI), the therapeutic approaches for AKI are disappointing. This is largely attributed to the poor understanding of the pathogenesis of AKI. Heparanase (Hpa), an endoglycosidase that cleaves heparan sulfate, is involved in extracellular matrix turnover, inflammation, angiogenesis, and cancer metastasis. Although an association between Hpa and glomerular diseases was demonstrated, a role for Hpa in the pathogenesis of ischemic AKI has not yet been elucidated.

## Objective

The current study examines the involvement of Hpa in the pathogenesis of ischemic reperfusion (I/R) AKI.

## Materials and Methods

Hpa transgenic mice (Hpa-tg) and their wild-type (wt) BALB/c mice were subjected to bilateral renal ischemia for 30 min followed by reperfusion. Two additional groups of wt and Hpa-tg mice were pretreated with PG545, a Hpa inhibitor. Mice were sacrificed after 48 and 72 h, and their blood and kidneys were collected. Serum creatinine (Scr) and blood urea nitrogen (BUN) were determined. The kidneys were examined for Hpa enzymatic activity, gene expression, immunofluorescence, and various fibrosis markers.

## Results

I/R induced AKI in both wt and Hpa-tg mice, as evident by typical tubular damage which was more profound in Hpa-tg mice. The latter also displayed exaggerated elevation in Scr and BUN. Moreover, TGF-β, vimentin, and α-smooth muscle actin (biomarkers of fibrosis), as well as TNFα, IL6, and ET-1 (biomarkers of inflammation), were upregulated in I/R-induced AKI, especially in the Hpa-tg mice. Pretreatment with PG545 abolished kidney dysfunction and histological changes induced by I/R.

## Conclusions

The present study provides new insights into the involvement of Hpa in the pathogenesis of ischemic AKI. Specifically, our results indicate that Hpa plays a deleterious role in the development of renal injury and kidney dysfunction characterizing this clinical setting. Moreover, Hpa inhibition comprises a novel therapeutic approach for AKI.

# Abstract: Second-opinion Consultation: Contributing or Confusing?

**Published:** 2017-01

**Authors:** Roni Gagin, Neta HaGani, Esti Zigelboim, Menashe Zaaroor

**Affiliations:** 1 Department of Social Work, Rambam Medical Center, Haifa, Israel; 2 Department of Neurology, Rambam Medical Center, Haifa, Israel

## Introduction

Second-opinion consultations are often required in complex medical situations in order to assist patients and families with decisions regarding further care. They are also used as a tool for doctors to share responsibility for patients in order to avoid negligence or insufficient treatment. In Israel, The Patient’s Rights Law (1996) insures the right to a second opinion. However, it is not clear to what extent patients are aware of this right, whether it is being used by patients and doctors during hospitalization, and if it is useful for patients.

## Objective

To identify factors associated with seeking second-opinion consultations during hospitalization.

## Materials and Methods

A structured questionnaire was completed by 91 patients and their families from the Neurosurgical Unit at Rambam Medical Center, Haifa, Israel. The questionnaire included items regarding: previous experience in seeking a second opinion, satisfaction from the consultation, reasons for not seeking a second opinion, and socio-demographic characteristics. The data analysis included correlations between seeking a second opinion and demographic characteristics and other factors that may be associated.

## Results

The study found that 79% of patients were unaware of The Patient’s Rights Law and their right to a second opinion consultation. Only 31% of patients reported accessing a second opinion regarding their current medical status, before or during hospitalization; 58% reported using second-opinion consultations for other medical conditions in the past. The main reason for not consulting a second opinion was full trust in the current doctor (48%). Amongst those who turned to a second opinion (for current or past medical conditions) only 7% reported that their doctor initiated the consultation, 54% of patients did not inform their first doctor about the results of the second consultation, while 64% indicated that the second opinion clarified their medical condition.

## Conclusions

When facing sudden and traumatic medical situations, patients tend to put their trust in the current doctor and find it difficult to consider other options. Patients might be worried about the reactions of the first doctor and may benefit from their doctor’s encouragement and assistance in requesting a second-opinion consultation. There is a need to empower patients and increase their access to medical information. It is important to raise awareness among patients and doctors in order to decrease liability and increase treatment compliance among patients.

# Abstract: Illness Severity and Psychosocial Variables as Correlates of Quality of Life in Adolescents Living with Inflammatory Bowel Disease

**Published:** 2017-01

**Authors:** Adi Pachter-Alt, Varda Soskolne, Ron Shaoul

**Affiliations:** 1 Rambam Medical Center, Haifa, Israel; 2 School of Social Work, Bar Ilan University, Ramat Gan, Israel

## Introduction

The number of adolescent patients with chronic inflammatory bowel disease (IBD) has risen over the past decades. These chronic diseases cause extended periods of physical morbidity and compromise the quality of life (QoL) of the adolescents and their families. Innovation in this study was a combination of variables from two theoretical models of adaptation to chronic illness: the Stress and Coping Model (by Lazarus and Folkman, 1984) and the Ecological Family Framework (by Pedersen and Revenson, 2005).

## Objectives

To examine correlates of QoL in adolescents living with IBD. Specifically, to examine the direct and indirect associations of the stressor (disease severity), cognitive appraisal (illness perception), coping resources (self-efficacy, positive affect, social support), and family resources (economic situation, family functioning) with QoL of the adolescent living with IBD.

## Materials and Methods

A total of 74 dyads of adolescents (44 boys and 30 girls) living with IBD (11–18 years old, patients of the Pediatric Gastroenterology Clinic, Rambam Medical Center) and their mothers were included. The adolescents completed a structured questionnaire measuring cognitive appraisal, coping resources, and QoL. The mothers completed a questionnaire on family resources. Physicians assessed illness severity.

## Results

Disease severity was significantly associated with the adolescents’ QoL but was secondary to the contribution of the subjective illness perception to QoL: over half of the variance in QoL was explained by illness perception (the higher it was perceived as threatening, the worse the QoL), followed by a significant yet weak contribution of disease severity and social support. Additionally, social support moderated the association between disease severity and QoL and the association between illness perception and the adolescents’ QoL. At all levels of social support, a negative significant association was found between illness perception and QoL, but the association was stronger at the lowest levels of social support. Other resources and background variables were not significantly related to adolescents’ QoL.

## Conclusions

Illness severity is not sufficient to explain QoL, and its contribution is secondary to the adolescents’ perception of the disease. In addition, the adolescents’ personal social support but no other personal or family coping resource contributed to QoL. These findings suggest that the medical team should frequently convey information about the disease and treatments in a way fully adapted to the adolescents’ understanding, in order to decrease perception of the illness as threatening, and find ways to enhance the adolescents’ social support.

# Abstract: The Effect of Gamma-Sterilization on the Antibacterial Function and Properties of Hybrid Wound Dressing

**Published:** 2017-01

**Authors:** Ayelet Raz-Pasteur, Eytan Mazor, Israela Berdicevsky, Maoz Shemesh, Meital Zilberman

**Affiliations:** 1 Department of Microbiology, Ruth & Bruce Rappaport Faculty of Medicine, Technion–Israel Institute of Technology, Haifa, Israel; 2 Department of Internal Medicine A, Rambam Medical Center, Haifa, Israel; 3 Infectious Diseases Unit, Rambam Medical Center, Haifa, Israel; 4 Department of Biomedical Engineering, Tel Aviv University, Tel Aviv, Israel

## Introduction

A new bioresorbable hybrid wound dressing which combines a synthetic porous top layer with a spongy collagen sublayer was developed and studied. The top layer contains the antibiotic drug gentamicin for controlled release to the wound site. It is of very high importance to use an appropriate sterilization process that will not have a deleterious effect on the function of this new wound dressing.

## Materials and Methods

Our investigation focused on the effects of gamma-irradiation sterilization (10, 25, 35, and 50 kGy), performed at room temperature and in liquid nitrogen, on the antibacterial efficacy and on the mechanical and physical properties of this wound dressing.

## Results

The gentamicin release profile from the hybrid wound dressing was very effective against three relevant bacterial strains for a minimum of 14 days: *Staphylococcus albus*, *Staphylococcus aureus*, and *Pseudomonas aeruginosa*. The effectiveness did not decrease after gamma-irradiation sterilization, even at high irradiation doses. The changes in the mechanical and physical properties, which were probably due to a combination of crosslinking and chain scission of the hybrid layers by the gamma radiation, were even beneficial when radiation doses were in the range of 10–35 kGy.

## Conclusions

It can be concluded that gamma-irradiation is a desired method of sterilization for our novel antibiotic-eluting hybrid wound dressing.

# Abstract: Internal Ward Falls: Retrospective Characteristics Study

**Published:** 2017-01

**Authors:** Joshua Aviram, Yaffa Zelig, Yosra Diab, Inna Tzoran, Amir Karaban

**Affiliations:** Internal Ward C, Rambam Medical Center, Haifa, Israel

## Introduction

A literature review on falls in hospitalized patients indicates serious widespread problems. Furthermore, fall rates are much higher in elderly care and rehabilitation units. Fall events may cause psychological trauma, loss of independence, or even death. Moreover, the financial cost of falling is enormous both to the individual for physical and psychological services, and to health services for resources and bed occupancy.

## Objective

The study retrospectively analyzed fall reports during January 2015–April 2016, at the Internal Ward C, Rambam Medical Center in Israel, in order to identify specific risk factors for fall events.

## Materials and Methods

Data were extracted from original fall event reports and electronic medical records. Descriptive statistics were generated for all outcomes. Relationships between fall events characteristics were examined using Pearson correlation tests.

## Results

Seventy-two fall reports were analyzed. Patients’ age was 62.2±12.8; most (72.9%) were described as “fully oriented in time and place.” Patient diagnoses included hypertension (80.6%), malignancies (45.8%), diabetes (41.7%), cerebrovascular disease (15.3%), and chronic obstructive pulmonary disease (COPD) (19.4%). Patients took 7.4±4.6 medications, including: hypnotics (40.3%), sedatives (31.9%), diuretics (30.6%), and opiates (16.7%). Most (30, 41.7%) fall events occurred during the night shift (23.00–07.00), 24 (33.3%) falls occurred during the morning shift (07.00–15.00), and 18 (25.0%) during the evening shift (15.00–23.00). Most fall events (55.6%), occurred in the patient’s room, where in 18 (25.0%) events the fall occurred during patients’ attempt to self-transfer their position; 28 (38.9%) of falls occurred at the shower due to “slipping.” Thirty-six (50.0%) fall events caused external injury; however, none of the events directly caused death. Notably, 17 (23.6%) patients died during the six-month period following the fall event. Correlation tests indicated that older patients received higher fall scale scores and degrees (*P*<0.05 and *P*<0.0001, respectively). Furthermore, lower percentages of capillary oxygen were associated with higher incidences of injury (*P*<0.05).

## Conclusions

Most reported falls occurred in oriented patients without dementia, mostly at night, and under extensive hypnotic medications treatment. Interestingly, mortality at six months post fall event during hospitalization were reported for 25% of the sample. These findings may be useful for future interventions to reduce fall rates in internal medicine wards.

# Abstract: A Multi-Center Registry of Medical Cannabis (MC) for Chronic Pain: Initial Results

**Published:** 2017-01

**Authors:** Joshua Aviram, Dorit Pud, Elon Eisenberg

**Affiliations:** 1 Faculty of Social Welfare and Health Sciences, University of Haifa, Israel; 2 Internal Ward C, Rambam Medical Center, Haifa, Israel; 3 Institute of Pain Medicine, Rambam Medical Center, Haifa, Israel; 4 The Ruth & Bruce Rappaport Faculty of Medicine, Technion–Israel Institute of Technology, Haifa, Israel

## Introduction

Although the use of medical cannabis (MC) for chronic pain is rising, scientific evidence for its long-term efficacy is scarce. A multi-center registry was structured in late 2015 to prospectively collect data on the effectiveness and safety of MC use in Israel. This study presents an initial report of patients’ baseline characteristics and short-term effects of MC.

## Objective

To identify predictors for success/failure of MC treatment.

## Materials and Methods

Ethics committees relevant to participating physicians approved the study. Patients were recruited by pain specialists, following completion of a new application for MC use (submitted to the Israel Ministry of Health). A written informed consent was obtained from applicants, followed by web-based data collection (Qualtrics software) at baseline and at 1, 3, 6, 9, and 12 months following initiation of MC use. Data included demographics, pain diagnoses and treatment, concomitant medical conditions, and self-completed questionnaires, including: pain intensity (NPS, 0–10; and SF-MPQ, 0–45), Pain Disability Index (PDI, 0–10), Pain Catastrophizing Scale (PCS, 0–52), Pittsburgh Sleep Quality Index (PSQI, 0–21), General Anxiety Disorder (GAD-7, 0–21), Beck Depression Inventory (BDI, 0–63), and Quality of Life (EQ5, 0–10). A paired *t* test was used to compare baseline and follow-up scores.

## Results

Baseline characteristics included 384 patients (252 men) with a mean age of 49±16 years; 52% were employed/students and 48% were retired/unemployed. Neuropathic pain was the most common diagnosis (71%), followed by musculoskeletal pain (51%), dysfunctional pain (14%), headache (13%), visceral pain (13%), and cancer pain (1%). Some patients had concomitant diagnoses. To date, characteristics for 136 patients (74 men) have been obtained at one-month follow-up. Most patients received 20 g of MC by inhalation (*n*=108) or as oil extracts (*n*=25). A significant (*P*<0.0001) drop (=improvement) was noted in all parameters. Regarding pain intensity, NPS decreased from 8.0±1.5 to 6.6±2.1, and SF-MPQ decreased from 25.19±9.0 to 20.2±10.2. Indicator scores decreased for disability (PDI from 6.6±1.7 to 5.4±2.2), catastrophizing (PCS from 37.7±10.8 to 33.7±13.2), sleep quality (PSQI from 10.8±3.4 to 7.1±3.4), anxiety (GAD from 10.4±6.5 to 6.7±6.0), depression (BDI from 20.6±10.9 to 14.7±10.2), and quality of life (EQ5 from 4.9±1.7 to 3.9±1.8). Fifty-eight patients reported adverse events, mostly mild.

## Conclusions

Candidates for MC treatment have significant pain and disabilities. Preliminary findings suggest a short-term improvement of pain and associated symptoms by MC.

# Abstract: The Use of Matrix-assisted Laser Desorption Ionization–Time of Flight Mass Spectrometry (MALDI-TOF-MS) for Rapid Bacterial Identification in Patients with Smear Positive Bacterial Meningitis—a Study of Diagnostic Accuracy

**Published:** 2017-01

**Authors:** Boaz Bishop, Yuval Geffen, Ola Kassis, Mical Paul, Ami Neuberger

**Affiliations:** 1 Department of Internal Medicine B, Rambam Medical Center, Haifa, Israel; 2 Microbiology Laboratory, Rambam Medical Center, Haifa, Israel; 3 Unit of Infectious Diseases, Rambam Medical Center, Haifa, Israel; 4 Ruth & Bruce Rappaport Faculty of Medicine, Technion–Institute of Technology, Haifa, Israel

## Introduction

Bacterial meningitis is a rapidly occurring fatal disease, thus immediate diagnosis and adequate therapy are crucial. Diagnosis is obtained by cerebral spinal fluid (CSF); cultures results are the gold standard for diagnosis, but are available only within 24–72 hours. Matrix-assisted laser desorption ionization–time of flight mass spectrometry (MALDI-TOF-MS) identifies a wide range of bacteria by examining protein profiles. Data are scarce regarding the use of MALDI-TOF-MS for examining CSF samples. We aimed at assessing the accuracy of MALDI-TOF-MS in rapid identification of CSF pathogens in patients with meningitis.

## Materials and Methods

We conducted a prospective study examining samples with positive CSF Gram stains with MALDI-TOF-MS. The results were compared with the CSF culture; identification was considered accurate when identical to the CSF culture results (species and genus level).

## Results

MALDI-TOF-MS was tested on 41 CSF samples with positive Gram stain: 17 cases of Gram-positive cocci, and 24 cases of Gram-negative bacteria. Nine patients had community-acquired meningitis, and 32 patients had post-neurosurgical meningitis. The technique was found to have a sensitivity of 80% and positive pressure ventilation (PPV) of 91% in examining Gram-negative CSF infections. Additionally, MALDI-TOF-MS correctly identified 2/5 samples with negative Gram stain. Only one Gram-positive coccus (*Staphylococcus aureus*) was identified by MALDI-TOF-MS.

## Conclusions

In CSF samples, MALDI-TOF-MS was relatively sensitive and specific in rapid identification of Gram-negative rods, but not for Gram-positive cocci. MALDI-TOF-MS may be used for a more rapid identification of Gram-negative bacteria in CSF samples, to aid in modifying the empiric treatment administered to these hospital-acquired pathogens, which are often multi-drug resistant.

# Abstract: Acute Kidney Injury after Primary Angioplasty: Is Contrast-Induced Nephropathy the Culprit?

**Published:** 2017-01

**Authors:** Oren Caspi, Manhal Habib, Yuval Cohen, Jennifer Le’or Fathy, Arthur Kerner, Ariel Roguin, Eitan Abergel, Monther Boulos, Robert Dragu, Michael R. Kapeliovich, Rafael Beyar, Eugenia Nikolsky, Doron Aronson

**Affiliations:** Department of Cardiology, Rambam Medical Center, Haifa, Israel; and the Ruth & Bruce Rappaport Faculty of Medicine, Technion–Israel Institute of Technology, Haifa, Israel

## Introduction

Acute kidney injury (AKI) following primary percutaneous coronary intervention (PCI) is frequently interpreted as contrast-induced AKI. However, in the context of ST-elevation myocardial infarction (STEMI), AKI may be the result of other contributing factors.

## Materials and Methods

We studied 2,200 STEMI patients who underwent primary PCI, and 1,025 patients receiving fibrinolysis or no reperfusion (control group).

## Results

AKI rates (defined as a creatinine rise of ≥0.5 mg/dL or >25% within 72 h following contrast exposure) were similar for the primary PCI and control group (10.3% vs. 12.1%, respectively; *P=* 0.38). In the PCI cohort, independent predictors of AKI included age ≥70, insulin-treated diabetes, diuretic therapy, anterior infarction, baseline estimated glomerular filtration rate; and variables related to the presence of pump failure (higher Killip class and use of intra-aortic balloon pump) and reduced left ventricular ejection fraction, but not contrast-medium dose. A risk score based on the PCI cohort indicated a similar discriminatory capacity for AKI in the PCI group and the control group (c-statistic 0.81±0.02 and 0.78±0.02, respectively; *P=*0.26). A mixed model demonstrated that the daily increase in creatinine with primary PCI was 0.042 mg/dL (95% confidence level (CI), 0.027–0.057) and 0.027 mg/dL (95% CI, 0.015–0.039) for the control group (*P=*0.44).

## Conclusions

The development of AKI in STEMI undergoing primary PCI is mainly related to older age, baseline eGFR, heart failure, and hemodynamic instability. Risk for AKI is similar amongst STEMI patients with and without contrast medium exposure.

# Abstract: Using a Smartphone App with a Reinforcement-Learning-Algorithm Generating Personalized Messages to Encourage Physical Activity in Sedentary Patients with Type 2 Diabetes

**Published:** 2017-01

**Authors:** Irit Hochberg, Mark Kozdoba, Guy Feraru, Shie Mannor, Moshe Tennenholtz, Elad Yom-Tov

**Affiliations:** 1 Institute of Endocrinology, Diabetes and Metabolism, Rambam Medical Center, Haifa, Israel; 2 Faculty of Electrical Engineering, Technion–Israel Institute of Technology (IIT), Haifa, Israel; 3 The Ruth & Bruce Rappaport Faculty of Medicine, Technion–IIT, Haifa, Israel; 4 Faculty of Electrical Engineering, Technion–IIT, Haifa, Israel; 5 Faculty of Industrial Engineering and Management, Technion–IIT, Haifa, Israel; 6 Microsoft R&D Center, Herzliya, Israel

## Introduction

Type 2 diabetes is the pandemic of the current era. One reason for the dramatic increase in type 2 diabetes prevalence is lack of regular physical activity. Most individuals with type 2 diabetes are sedentary, although they and their health care providers are well aware of the benefits of being physically active. We hypothesized that continuous communication and monitoring of patients through their smartphone could promote physical activity, and that a computer algorithm could select the best messages to encourage activity in each individual based on their responses to previous messages.

## Materials and Methods

We advised 27 sedentary type 2 diabetes patients to walk regularly and installed an app in their smartphone to monitor and record their activity. The patients were divided into two groups: (1) Personalized treatment group receiving personalized SMS messages generated by an automatic reinforcement-learning algorithm, which optimized messages to encourage physical activity for each participant; and (2) Control group receiving a static weekly SMS message with a reminder to exercise.

## Results

The participants in the personalized treatment group increased their amount of activity (weekly slope +0.012±0.002) and walking rate (weekly slope +0.02±0.005), while the control group patients did not (weekly slope −0.004± 0.002 for activity and −0.01±0.007 for walking rate). Extended participation in the trial with allocation to the learning reinforcement algorithm group led to improvement in HbA1c (*P*=0.02). The learning algorithm gradually improved its ability to predict messages that led to increased exercise for participants.

## Conclusions

The results of this small-scale study provide the first evidence of the novel concept that a learning algorithm can coach humans. For patients with diabetes, improved adherence to exercise resulted in improved glycemic control. Implementing this concept has the potential for meaningful clinical results on a large scale, as an automatic personalized learning algorithm could be used in large populations to improve health and glycemic control.

# Abstract: Pregnancy and Its Related Hormones in Promoting Non-Hodgkin Lymphoma

**Published:** 2017-01

**Authors:** Tami Katz, Ali Abd El Wahed, Noam Bettman, Michal Hayun, Netanel A. Horowitz

**Affiliations:** 1 Department of Hematology and Bone Marrow Transplantation, Rambam Medical Center, Haifa, Israel; 2 Ruth & Bruce Rappaport Faculty of Medicine, Technion–Israel Institute of Technology, Haifa, Israel

## Introduction

Lymphoma is the most common hematological cancer reported during pregnancy. Recent data suggest that unlike lymphoma occurring outside of pregnancy, pregnancy-associated non-Hodgkin lymphoma is characterized by an excessive involvement of reproductive organs, advanced disease stage at diagnosis, and an aggressive course. However, the mechanisms facilitating this clinical phenomenon are not fully defined.

## Objective

The current study aimed to explore the hypothesis that the pregnancy-induced hormonal milieu could be an important mediator in the interaction between lymphoma cells and their associated microenvironment, which may potentially contribute to the growth and progress of lymphoma developing during pregnancy.

## Materials and Methods

BALB/c pregnant and non-pregnant mice were subcutaneously inoculated with murine B cell lymphoma cells (A20). Tumor growth was measured biweekly in both groups. Human lymphoma cell lines (Ramos, Raji, and BL-2) and the lymph node (LN) stromal cell line (HK) were assessed using flow cytometry, Western blot, and qPCR analysis for the expression of estrogen and progesterone receptors (ER α&β and PR). The direct effect of estradiol (E2) or progesterone on lymphoma cell proliferation was analyzed by trypan blue exclusion. The effect of E2 on the expression of growth factors VEGF-C and VEGF-D and their receptor VEGFR3 in lymphoma and stromal cells was analyzed by qPCR. Similarly, the expression of metalloproteinases MMP-2 and MMP-9 was evaluated.

## Results

Pregnant mice showed a significantly accelerated tumor volume and weight following lymphoma cell inoculation, compared to non-pregnant mice. All lymphoma cell lines and LN stromal cells expressed ER α&β, but not PR. The Ramos cell line treated with E2 demonstrated an increased proliferation rate. This effect was found to be mediated by ERα. In contrast, progesterone had no effect on lymphoma cell proliferation or viability. Furthermore, E2 induced the expression of VEGF-C and VEGF-D mRNA in lymphoma and LN stromal cells. Notably, the expression of their receptor VEGFR3 was elevated in lymphoma cells. E2 treatment resulted in increased expression of MMP-2 and MMP-9 mRNA in lymphoma and LN stromal cells, respectively.

## Conclusions

This study demonstrated for the first time that pregnancy significantly enhances lymphoma growth *in vivo*. This finding could be partially explained by a direct impact of pregnancy-induced estrogen on lymphoma cell proliferation, through its effect on the expression of growth factors and metalloproteinases on lymphoma and LN stromal cells.

# Abstract: Heparanase in Acute Pancreatitis: New Insights into Pathogenesis and Therapy

**Published:** 2017-01

**Authors:** Iyad Khamaysi, Preeti Singh, Neta Ilan, Israel Vlodavsky, Alessandro Noseda, Hoda Awad, Yehuda Chowers, Zaid Abassi

**Affiliations:** 1 Department of Gastroenterology, Rambam Medical Center, Haifa, Israel; 2 Cancer and Vascular Biology, Rambam Medical Center, Haifa, Israel; 3 Sigma-Tau Research, Mendrisio, Switzerland; 4 Research Unit, Rambam Medical Center, Haifa, Israel; 5 Department of Physiology, The Ruth & Bruce Rappaport Faculty of Medicine, Technion–Israel Institute of Technology, Haifa, Israel

## Introduction

Despite advances in understanding the pathogenesis of acute pancreatitis (AP), the mechanisms underlying this disease have not been fully determined. In the majority of cases, AP is a self-limited process, yet 20% of patients develop a severe form of AP with pancreatic necrosis, multi-organ involvement, and high mortality. Heparanase (HPSE), an endoglycosidase which cleaves heparan sulfate, degrades and remodels the extracellular matrix. Heparanase is preferentially expressed in human tumors, including pancreatic adenocarcinoma. While the role of HPSE in cancer has been extensively studied, the involvement of this enzyme in inflammation, and in AP in particular, remains obscure.

## Objective

The current study examines if HPSE is involved in the pathogenesis of cerulein-induced AP in mice.

## Material and Methods

Heparanase-over-expressing transgenic mice (*hpa*-TG) and wild-type (WT) BALB/c mice were intraperitoneally injected with either cerulein (50 mg/kg, 5 times, at 1 hour apart) or vehicle, with or without low and high doses of roneparstat (SST0001, HPSE inhibitor) pretreatment. The animals were sacrificed 24 hours following the development of pancreatitis. The pancreatic response and the severity of AP were evaluated by pancreatic HPSE activity (determined by Na_2_^35^SO4-labeled extracellular matrix (ECM)), pancreatic edema index (determined by organ to animal weight ratio), tissue inflammatory response (determined by histopathological analysis), autophagy response (determined by electron microscopy and immunohistochemistry staining), and serum pancreatic enzymes (amylase and lipase) levels.

## Results

Cerulein-induced AP in wild-type mice was associated with significant rises in the serum levels of amylase and lipase. These increases were characterized by an enhancement of HPSE activity, a higher pancreatic edema index, and tissue inflammation and autophagy response. All types of responses to administration of cerulein were profoundly exaggerated in *hpa*-TG mice. In contrast, when cerulein was injected to heparanase knockout (*hpa*-KO) mice, the severity of pancreatic injury was attenuated as compared with their wild-type controls. Importantly, pretreatment with roneparstat significantly reduced, in a dose-related manner, the HPSE activity, the tissue inflammatory response, autophagy, and serum amylase and lipase levels.

## Conclusions

HPSE appears to play an important role in the pathogenesis of AP. The HPSE inhibitor (roneparstat) significantly reduced the severity of the AP in an animal model. This new concept may provide a basis for prophylaxis and treatment of AP.

# Abstract: Rheological Analysis of Pancreatic Cyst Fluid Accurately Differentiates Pancreatic Cyst Types

**Published:** 2017-01

**Authors:** Iyad Khamaysi, Gleb Vasilyev, Aiman Abu Ammar, Yehuda Chowers, Eyal Zussman

**Affiliations:** 1 Department of Gastroenterology, Rambam Medical Center, Haifa, Israel; 2 Faculty of Mechanical Engineering, Technion–Israel Institute of Technology (IIT), Haifa, Israel; 3 The Ruth and Bruce Rappaport Faculty of Medicine, Technion–IIT, Haifa, Israel

## Introduction

Pancreatic mucinous type cysts have the potential for malignant transformation. Differentiation between benign and potentially malignant pancreatic cysts is exceedingly important yet remains difficult. The relative viscosity of pancreatic cyst fluid has proved useful for distinguishing mucinous from non-mucinous cysts and is an indirect measure of cyst fluid mucin, glycoproteins, and DNA content. In previous studies, the viscosity was assessed by the string sign (elongation viscosity), a surrogate marker of cyst fluid viscosity. However, the method lacks a theoretical framework for predicting the viscoelastic nature of the cyst fluid, a rheological property of complex physiologic fluids having two components, viscosity and elasticity.

## Objective

To assess the utility of the rheological properties (measured by a rheometer) of pancreatic cyst fluid, as compared with cytology and biochemical analysis in differentiating pancreatic cyst types.

## Materials and Methods

Endoscopic ultrasound (EUS)/fine needle aspiration (FNA) EUS-FNA was performed on consecutive subjects with pancreatic cysts. In addition to routine cyst fluid analyses (biochemical, cytological, and viscosity assessment using the string sign), a rheological behavior curve of the cyst fluid was generated (steady-state shear viscosity at different shear rates measured with a highly sophisticated rheometric apparatus). Pancreatic cysts were classified as mucinous (mucinous cystadenoma, mucinous adenocarcinoma, intraductal papillary mucinous neoplasm (IPMN)) or non-mucinous (serous cystadenoma, pseudocyst) based on surgical and/or clinical out-come (presentation, follow-up, imaging, and fluid analyses).

## Results

Fifteen subjects were evaluated (9 females, median age 53 years). Mean cyst size was 33.1 mm (range 20–60 mm). Six mucinous and 9 non-mucinous cysts were diagnosed. Four mucinous cysts were diagnosed surgically. Median follow-up was 18 months.

Three different patterns of rheological behavior curves were identified (Figure 1): pattern I (constant viscosity <1.6 units), pattern II (rapidly decreasing viscosity to <1.6 units), and pattern III (slowly decreasing viscosity >1.6 units). Patterns I and II correlated with non-mucinous cysts, and pattern III correlated with mucinous cysts. This curve pattern analysis was evaluated as a new diagnostic test.

**Figure 1:** Khamaysi et al. Rheological Results; Viscosity vs. Shear Stress.

The concordance between the string sign and the measured relative viscosity was low (*P*=0.62, chi-square). The sensitivity, specificity, positive and negative predictive values and accuracy of string sign, elevated carcinoembryonic antigen (CEA) (>192 ng/mL), cytology, and rheology (curve patterns I and II, non-mucinous; curve pattern III, mucinous) are presented in Table 1.

**Table 1:** Khamaysi et al. The Sensitivity, Specificity, Positive, and Negative Predictive Values and Accuracy of the Various Diagnostic Methods.

	String Sign	CEA	Cytology	Rheology
Sensitivity	0.67	0.67	0.5	1
Specificity	0.67	0.89	1	0.89
PPV	0.57	0.8	1	0.86
NPV	0.75	0.8	0.75	0.83
Accuracy	0.67	0.8	0.8	0.93
*P* Value	0.32	0.09	0.05	0.001

Non-mucinous cysts can be further differentiated according to observed rheological curve patterns (pattern I, serous; pattern II, inflammatory pseudocysts, data not shown).

## Conclusion

The accuracy of the string sign, CEA, and cytology for mucinous cysts are comparably inadequate. Cyst fluid rheological analysis appears accurately to differentiate amongst pancreatic cyst types.

# Abstract: The Effect of Oseltamivir Treatment on the Yield of Polymerase Chain Reaction Test for Confirmed Influenza Infection Among Adults: A Prospective, Cohort Study

**Published:** 2017-01

**Authors:** Johad Khoury, Maram Saffuri, Khadeje Seh, Moran Szwarcwort, Zipi Kra-oz, Eyal Braun, Zaher Azzam, Michal Paul, Ami Neuberger

**Affiliations:** 1 Internal Medicine Department B, Rambam Medical Center, Haifa, Israel; 2 The Ruth & Bruce Rappaport Faculty of Medicine, Technion–Israel Institute of Technology, Haifa, Israel; 3 Virology Laboratory, Rambam Medical Center, Haifa, Israel; 4 Internal Medicine Department H, Rambam Medical Center, Haifa, Israel; 5 Department of Infectious Diseases, Rambam Medical Center, Haifa, Israel

## Introduction

Influenza is caused by influenza viruses that usually infect the upper and the lower respiratory tract.

The clinical diagnosis of influenza has modest sensitivity (64%–65%) and specificity (67%). Laboratory tests are therefore needed in order to confirm the diagnosis. The gold standard for the diagnosis is reverse transcriptase polymerase chain reaction (RT-PCR).

PCR tests are often undertaken with delays of hours to days after initiating treatment, because of the low availability of these tests. Theoretically, this delay may lead to false negative PCR results, leading to unnecessary antibiotic administration for suspected bacterial infections.

## Objective

To determine the rate of decline in the diagnostic yield of influenza PCR assay after oseltamivir administration, and to identify risk factors for prolonged shedding of the virus.

## Materials and Methods

The study population included patients older than 18 years with clinical signs of influenza admitted to Rambam Medical Center during the 2015 and 2016 influenza seasons, with positive influenza PCR tests, who had been treated with oseltamivir. Clinical follow-up and repeat PCR testing were performed on days 2, 4, and 6 of treatment. We defined “prolonged shedding” as a positive PCR test on day 2.

## Results

The inclusion criteria were met by 214 patients, 105 males (49.1%). Median age for prolonged shedders was 65.5 (*n*=142, SD=17.3) and for non-shedders 63.9 (*n*=72, SD=63.9) years; 72 patients (33.6%) had negative PCR result at day 2, 28/78 patients (35.8%) were negative at day 4, and 11/31 (35.4%) were negative at day 6.

Both male gender and solid organ or bone marrow transplants are risk factors for prolonged shedding, *P*=0.036 and *P*=0.03, respectively.

## Conclusion

PCR testing after more than 48 hours of anti-viral treatment has a high rate of false positive tests (33.6%). Thus, a negative PCR test taken more than 48 hours after initiation of treatment should not lead the clinician to assume that the patient does not have influenza.

# Abstract: Secondary Antibiotic Resistance of *Helicobacter pylori* Isolates in Israeli Children and Adults

**Published:** 2017-01

**Authors:** Johad Khoury, Yuval Geffen, Ron Shaul, Hisham Sholy, Yehuda Chowers, Tarek Saadi

**Affiliations:** 1 Liver Unit, Rambam Medical Center, Haifa, Israel; 2 Department of Internal Medicine B, Rambam Medical Center, Haifa, Israel; 3 Microbiology Laboratory, Rambam Medical Center, Haifa, Israel; 4 Pediatric Gastroenterology Unit, Rambam Medical Center, Haifa, Israel; 5 Ruth & Bruce Rappaport Faculty of Medicine, Technion–Israel Institute of Technology, Haifa, Israel; 6 Department of Gastroenterology, Rambam Medical Center, Haifa, Israel

## Introduction

Failure of standard therapy for *Helicobacter pylori* infections result primarily from increasing antibiotic resistance. Patients in Israel are referred for *H. pylori* culture after failure of at least two therapeutic regimens. ***Objectives:*** To estimate the prevalence of secondary antimicrobial resistance of *H. pylori*.

## Materials and Methods

During 2012–2015, results of *H. pylori* cultures were retrospectively collected when gastric biopsies were performed at Rambam Medical Center, Haifa, Israel. Antimicrobial susceptibility to five drugs was tested by gradient diffusion.

## Results

During the study period, 107 patients (46 adults and 61 children) were referred for performance of *H. pylori* cultures. Cultures were positive in 64 samples (63.7%). In adults, 23 (50%) patients had positive *H. pylori* cultures; 8.69% showed resistance to amoxicillin (AM), 39.1% to clarithromycin (CH), 61.9% to metronidazole (MZ), 8.69% to tetracycline (TC), and 21.7% to levofloxacin (LEV). In children, 41 (67%) patients had positive *H. pylori* cultures; 5.1% showed resistance to AM, 42.5% to CH, 46.66% to MZ, 2.5% to TC, and 0% to LEV. In children, 94.9% of *H. pylori* strains were susceptible to both AM and LEV; in adults, 82.6% of the strains were susceptible to both AM and TC; and 28.6% of adults and 24.1% children were resistant to both MZ and CH.

## Conclusions

The sensitivity of *H. pylori* culture is low. The resistance of *H. pylori* to MZ and CH is very high after failure of two therapeutic regimens in both adults and children. No LEV resistance was detected in children. AM resistance was higher in adults than in children.

# Abstract: Norton Scale as a Predictor for 30-day Hospital Readmission Among Older Acute Medical Patients

**Published:** 2017-01

**Authors:** Dani Kirshner, Tzvi Dwolatzky

**Affiliations:** 1 Geriatric Unit, Rambam Health Care Campus, Haifa, Israel; 2 The Ruth & Bruce Rappaport Faculty of Medicine, Technion–Israel Institute of Technology, Haifa, Israel

## Introduction

Older people are at risk for hospital readmissions, and minimizing readmissions may improve quality of care and reduce health care costs. While the Norton scale was devised to predict the likelihood of pressure sore development, it is useful for predicting length of hospital stay, in-hospital complications, and mortality in older patients. We aimed to determine the value of the total Norton scale score and its component domains as a simple tool for predicting readmission within 30 days in older patients admitted to acute internal medicine wards.

## Materials and Methods

A 6-year retrospective study was performed based on the electronic records of medical inpatients aged 65 years and older at the Rambam Health Care Campus, with the primary outcome being readmission within 30 days. Data collected included: Norton scale on admission, patient demographics, initial admission ward, length of stay, and laboratory parameters. Bivariate and multivariate logistic regression analyses were conducted to predict risk of readmission.

## Results

Data analyzed comprised 34,329 hospitalizations, of which 18,044 (53%) were women with a mean age of 78.5±7.8 years. Mean primary hospitalization length was 7.0 days, and the 30-day readmission rate was 11.3%. Based on multivariate logistic regression analysis, scores of 2/4 and 1/4 for the level of activity component of the Norton scale were associated with increased risk for 30-day readmission (adjusted odds ratio [OR]: 1.33 and 1.64 respectively, 95% confidence interval [CI]: 1.22–1.45 and 1.49–1.8, respectively, *P*<0.001).

## Conclusions

The level of activity according to the Norton scale on admission is a useful measure for predicting the risk of 30-day readmission amongst older medical patients in an acute-care setting.

# Abstract: Health Care Disparities in Colorectal Cancer Early Detection and Prevention: Association with Patient Activation Measurements and Mother Tongue

**Published:** 2017-01

**Authors:** Jesse Lachter, Sarit Balanson, Hanna Lupo-Gralnic, Yakov Fogelman

**Affiliations:** 1 Gastroenterology Department, Rambam Medical Center, Haifa, Israel; 2 Ruth & Bruce Rappaport Faculty of Medicine**,** Technion–Israel Institute of Technology, Haifa, Israel; 3 Meuhedet Health Services, Haifa, Israel; 4 Leumit Health Services, Haifa, Israel

## Introduction

Cultural sensitivity in health care settings can lead to increased patient activation and aim at improving colorectal cancer (CRC) prevention. Patient activation is defined as “an individual’s knowledge, skill and confidence for managing his/her own health care” where the patient can “participate as an active member of the care team.”

## Objective

This study aimed to identify and assess disparities in colorectal cancer prevention and early detection, in association with measured levels of patient activation amongst Israelis stratified by mother tongue.

## Materials and Methods

Patients were surveyed from three outpatient clinics using the validated questionnaire Hibbard’s Patient Activation Measurement, utilized in 16 countries. The questionnaire was filled by 200 consenting patients aged >50 years independently or with assistance when needed.

## Results

Patient Activation Measurement Score (PAM Score) varied significantly according to gender, mother tongue, and years residing in Israel compared to native born Israelis. Women had a higher average PAM Score than men (61.5 vs. 57.2, *P*=0.04), indicating they reported being more participatory and active in self-care. Individuals with a mother tongue of Hebrew or Arabic had the highest PAM scores amongst the populations studied. Russian speakers had the lowest PAM scores overall (mean, 48.7) compared to native Hebrew speakers (*P*<0.0001). Individuals living in Israel for 11–20 years had significantly lower PAM Scores than native born Israelis (*P*=0.016), the difference thereafter diminishing. Populations with higher PAM Scores were more likely to undergo CRC screening.

## Conclusions

Significant variations in patient activation were found. The differences in PAM Scores indicate the need for more patient outreach, education, and policies that contribute to a more activated citizen. Limitations of the study included heterogeneous samples, availability of immigrant population subtypes, language barriers, incomplete questionnaires, and use of mother tongue to define status.

Next steps include developing and piloting interventions to enhance patient activation and prevent colon cancer.

# Abstract: Does Subjective Perception of “Pain Sensitivity” Correlate with Actual Sensitivity to Experimental Pain?

**Published:** 2017-01

**Authors:** Doron Meiselles, Joshua Aviram, Dorit Pud, Elon Eisenberg

**Affiliations:** 1 The Ruth & Bruce Rappaport Faculty of Medicine, Technion–Israel Institute of Technology, Haifa, Israel; 2 Institute of Pain Medicine, Rambam Medical Center, Haifa, Israel; 3 Faculty of Social Welfare and Health Sciences, University of Haifa, Haifa, Israel

## Introduction

People often state that they are “sensitive” or “insensitive” to pain. However, little is known about the relationships between people’s subjective self-perception of sensitivity to pain and their actual sensitivity to pain.

## Objective

To search for possible correlations between different components of subjective self-perception of sensitivity to pain and results of experimental pain measurements in healthy volunteers.

## Materials and Methods

The study included 84 healthy subjects (52 women). Subjective self-perception of sensitivity to pain included three variables: self-perceived “pain sensitivity”, self-perceived “pain threshold”, and subjective reported pain intensity in response to a hypothetical painful stimulus (bumping a toe into a solid object while walking fast), all measured on a scale of 0–10. Experimental pain (psychophysical measurements) consisted of thermal pain thresholds (°C), suprathreshold heat and cold stimuli (VAS 0–100), as well as conditioned pain modulation (CPM) and temporal summation (TS)—paradigms for testing inhibitory and excitatory pain processes, respectively. Pearson coefficient was used to test for correlations between self-reports of pain sensitivity and psychophysical measurements.

## Results

No significant correlations were found between self-perceived “pain sensitivity”, self-reported “pain threshold”, and any of the psychophysical measurements. In contrast, the reported pain intensity in response to the hypothetical painful stimulus correlated with the following parameters: cold pain threshold (*r*=0.249; *P*=0.023), suprathreshold cold pain intensity (*r*=0.304; *P*=0.005), and suprathreshold heat pain intensity (*r*=0.258; *P*=0.019) but not with CPM or TS.

## Conclusions

Self-perception of sensitivity to pain articulated by intangible expressions such as “pain threshold” or “pain sensitivity” is unrelated to actual sensitivity to experimental pain. However, a concrete notion such as the intensity of a hypothetical painful stimulus is associated with experimental pain reports and could potentially be utilized clinically. Self-reports are not directly related to the tested inhibitory or excitatory pain paradigms.

# Abstract: Increased Cardiac Troponin-I Is Associated with Averse Clinical Outcome in Patients with First Documented Episode of Acute Atrial Fibrillation

**Published:** 2017-01

**Authors:** Mohammad E. Naffaa, Roni Nasser, Elias Manassa, Maha Younis, Zaher S. Azzam, Doron Aronson

**Affiliations:** 1 Department of Internal Medicine “H”, Rambam Medical Center, Haifa, Israel; 2 The Ruth & Bruce Rappaport Faculty of Medicine, Technion–Israel Institute of Technology, Haifa, Israel; 3 Department of Internal Medicine “B”, Rambam Medical Center, Haifa, Israel; 4 Division of General Surgery, Rambam Medical Center, Haifa, Israel; 5 Division of Cardiology, Rambam Medical Center, Haifa, Israel

## Introduction

Recent-onset atrial fibrillation (AF) is a frequent cause for presentation to the emergency department. Recent studies proposed that the use of a myocardial injury biomarker might improve the capability to identify high-risk patients and, thus, predict clinical outcomes.

## Objective

We aimed to examine the role of serum cardiac troponin-I (cTnI) as a predictor of clinical outcome in patients with first documented episode of acute atrial fibrillation.

## Materials and Methods

Patients, 18 years or older, with a primary diagnosis of first documented episode of acute atrial fibrillation were included in this cohort. Variables associated with mortality or the composite end-point (mortality, stroke, or heart failure) in the univariate Cox regression analysis (Wald test; *P<*0.10) were included in the multivariable Cox model. The following clinical and laboratory data were considered in the multivariate procedure: age, gender, hypertension, diabetes mellitus, Charlson’s score, history of myocardial infarction, heart failure, stroke, peripheral vascular disease, serum creatinine, and elevated serum cTnI level.

## Results

Upon admission, 163 patients had normal cTnI levels, and 111 patients had elevated cTnI levels. Age >75 years, heart failure, Charlson’s score, hemoglobin level, and elevated cTnI were associated with increased mortality in the multivariate analysis.

## Conclusions

Cardiac troponin-I is a significant predictor of mortality and a composite end-point of mortality, stroke, or heart failure in patients presenting with the first documented episode of acute AF. Further prospective studies are needed to define its exact role in this setting.

# Abstract: Clinical Instability at Discharge in Acute Exacerbation of Chronic Obstructive Pulmonary Disease Is Associated with Hospital Readmission

**Published:** 2017-01

**Authors:** Roni Nasser, Rawan Azzam, Itai Gofman, Walid Saliba, Danny Epstein, Emilia Hardak, Zaher S. Azzam, Gidon Berger

**Affiliations:** 1 Department of Internal Medicine “B”, Rambam Medical Center, Haifa, Israel; 2 The Ruth & Bruce Rappaport Faculty of Medicine, Technion–Israel Institute of Technology, Haifa, Israel; 3 Department of Community Medicine and Epidemiology, Carmel Medical Center, Clalit Health Services, Haifa, Israel; 4 Division of Pulmonary Medicine, Rambam Medical Center, Haifa, Israel

## Background

Acute exacerbations of chronic obstructive pulmonary disease (AECOPD) are of major importance in terms of prolonged detrimental effects on patients, accelerated disease progression, and high health care costs. The goal of treatment in AECOPD is to minimize the impact of index exacerbation and prevent the development of subsequent exacerbations. The level of adherence to the hospital discharge criteria, as defined by the global initiative for chronic obstructive lung disease (GOLD) and its impact on readmission are unknown.

## Aim

To evaluate the adherence to the discharge criteria in “real life” and to examine whether it is predictive of readmission.

## Methods and Materials

This retrospective descriptive study included adult patients hospitalized at Rambam Health Care Campus (RHCC) for AECOPD between the years of 2011 and 2013. All patients were followed up for a year after discharge. According to the GOLD recommendations, four discharge criteria were examined, including clinical stability for 24 hours before discharge, arterial blood gases (ABG) stability for 24 hours before discharge, completion of follow-up arrangements, and treatment with and/or recommendation for long-acting bronchodilators prior to discharge. Accordingly, clinical and laboratory data were collected from all subjects. Each fulfilled criterion entitles one point, thus making up a 0–4 discharge score range. The adherence to the discharge criteria and the correlation between them and readmission were evaluated.

## Results

The 1,157 hospitalizations of 659 patients (mean age 69; males 62%) with AECOPD were reviewed. Forty-five patients died during hospitalization, hence 1,112 live discharges were analyzed. The 30-day and 60-day readmission rates for AECOPD were 15.47% and 23.11%, respectively. The degree of adherence to the GOLD recommended discharge criteria was 62%, 66%, 56%, and 50% for clinical stability, ABG stability, completion of follow-up arrangements, and treatment with and/or recommendation for long-acting bronchodilators prior to discharge, respectively. We found significant association between clinical and ABG stability at discharge, recommendation for long-acting bronchodilators in naïve patients, and early readmission.

## Conclusion

The adherence to the GOLD discharge criteria following hospitalization for AECOPD is only partial and significantly associated with early readmission. Consequently, novel approaches to improve adherence to and implementation of GOLD recommendations may have a beneficial effect on readmission rates.

# Abstract: Identifying the Presence and Content of Spiritual Distress in Israel: An Analysis of the Applicability of American Tools in Israel

**Published:** 2017-01

**Authors:** Michael Schultz, Rabbi, Tehilah Meged-Book, Tanya Mashiach, Gil Bar-Sela

**Affiliations:** 1 Division of Oncology, Rambam Medical Center, Haifa, Israel; 2 Ruth and Bruce Rappaport Faculty of Medicine, Technion–Israel Institute of Technology, Haifa, Israel; 3 Department of Statistics, Rambam Medical Center, Haifa, Israel

## Introduction

Spiritual distress is present in ~25% of oncology patients. Tools developed in the US to assess spiritual well-being serve to suggest directions for exploring the content of the distress, yet such tools cannot be used indiscriminately worldwide because the expression of spiritual distress likely shows cultural difference. This study aims to examine which subset of patients experience spiritual distress and to isolate culturally influenced elements of the Israeli expression of this universal experience. We also examined a general distress Visual Analogue Scale from 1 to 10 (Distress Thermometer) to determine if it suffices to identify spiritual distress.

## Materials and Methods

Structured interviews were conducted of oncology outpatients over 12 months, using a non-selective approach. Questions included the general distress thermometer and two key American tools for identifying the content of spiritual distress: FACIT-Sp-12 (composed of three subscales: Meaning, Peace, and Faith) and the Spiritual Injury Scale (SIS). Other questions concerned demographic, clinical, religious/spiritual, and social data.

## Results

Of 416 patients approached, 202 completed the interview (60% female, 82% Jewish, 58% metastatic disease); 23% reported spiritual distress. Significant predictors were patients’ self-evaluation of grave clinical condition (odds ratio [OR] 3.3, 95% CI 1.1–9.5) and patients’ perception of their condition as worse than the medical file indicated (OR 6.3, 95% CI 1.1–35.7). Of the FACIT-Sp-12 subscales, only Peace correlated with spiritual distress (receiver operating characteristic [ROC], area under curve 0.719). SIS as a whole strongly correlated (ROC, 0.765). A multivariate model, including all items individually, identified three parameters strongly predicting spiritual distress: not feeling peaceful, feeling sadness or grief, and feeling despair or hopelessness. The Distress Thermometer, though a significant predictor (OR 2.2, 95% CI 1.1–4.5) failed to identify most cases of spiritual distress (sensitivity 0.413).

## Conclusions

Spiritual distress is common among oncology patients, especially those who perceive—accurately or not—their clinical condition to be quite serious. The Distress Thermometer is not sufficient to identify spiritual distress. The cultural expression of spiritual distress in Israel differs in part from its expression in North America. The data suggest a model for an Israeli spiritual care intervention initially focusing on feelings of grief, despair, and non-peacefulness.

# Abstract: Evaluation of Microscopic Changes in Fallopian Tubes of Patients with Uterine Papillary Serous Carcinoma (UPSC) by Computerized Morphometric Analysis

**Published:** 2017-01

**Authors:** Amnon Amit, Edmond Sabo, Yamit Efrat Tamam, Geula Klorin

**Affiliations:** 1 Department of Obstetrics & Gynecology, Rambam Medical Center, Haifa, Israel; 2 Ruth & Bruce Rappaport Faculty of Medicine, Technion–Israel Institute of Technology, Haifa, Israel; 3 Department of Pathology, Rambam Medical Center, Haifa, Israel

## Introduction

Uterine papillary serous carcinoma (UPSC) is different in clinical and pathological behaviors from endometroid carcinoma, and more resembles ovarian cancer. Since there is evidence that indicates the origin of ovarian cancer is the fimbriae of the fallopian tubes, we aimed to evaluate changes of the fallopian tubes of patients with UPSC.

## Materials and Methods

A novel method of computerized morphometry of the fimbrial epithelium was used, in which a fast Fourier transformation (FFT) was applied to images of fimbrial epithelium, and the FFT two-dimensional frequency maps were subsequently quantified for nuclear orientation and planar distribution by a co-occurrence matrix analysis. Four morphometrics parameters were evaluated: homogeneity, contrast, correlation, and entropy. A total of 50 fimbriae reported as “normal” by H&E examination of patients with UPSC, endometrioid endometrial carcinoma (EEC), and healthy women were evaluated.

## Results

Significant differences were found between the fimbriae of patients with UPSC and patients with EEC, as well as between each one of them and the group of the healthy women.

## Conclusions

Using this novel method, we were able to demonstrate differences in morphometric characteristics of the fimbriae in healthy women and UPSC and EEC patients. It is yet to be determined whether these differences indicate that the UPSC originates from the fimbriae or is secondary to the uterine disease.

# Abstract: Complementary Treatment for Stress Reduction in Women with Ovarian Cancer—Assessment with Heart Rate Variability

**Published:** 2017-01

**Authors:** Amnon Amit, Lior Lowenstein, Keren Or Chen, Geula Korin, Amir Weissman

**Affiliations:** 1 Department of Obstetrics & Gynecology, Rambam Medical Center, Haifa, Israel; 2 Ruth and Bruce Rappaport Faculty of Medicine at the Technion–Israel Institute of Technology, Haifa, Israel; 3 Faculty of Social Welfare and Health Sciences, University of Haifa, Haifa, Israel

## Introduction

Following diagnosis of ovarian cancer, women are abruptly faced with fear of impending death, the burden of difficult treatments, concern regarding pain, and uncertainty about the future.

We aimed to assess the efficacy of anxiety reduction following the use of one mode of complementary and alternative medicine (CAM), “healing touch” therapy, in newly diagnosed women with ovarian cancer, using subjective and objective approaches.

## Materials and Methods

This is a prospective study in women diagnosed with ovarian cancer who had planned to undergo surgery. Complementary medicine in the form of healing touch therapy was offered to the women. Subjective feelings before and after treatment were documented by a questionnaire. Computerized electrocardiogram was performed before and following treatment. Analyses of heart rate variability was determined by power spectral density in order to study the autonomic nervous system modulation of heart rate, and non-linear analysis methods were used to explore “hidden” components of heart rate variability.

## Results

Significant decreases in tension, agitation, scare, panic, anxiety, and nervousness scores (*P*<0.001) and highly significant increases in relaxation, comfort, and tranquility scores (*P*<0.001) were found in all women according to the self-reported questionnaires. These were associated with a significantly decreased heart rate and the low-frequency band of the power spectral density, with a significant decrease in the short-term α1 slope (*P*<0.05) of the detrended fluctuation analysis.

## Conclusions

Subjective improved feelings reported by the women following healing touch therapy were corroborated by significant changes in heart rate variability, complexity, and fractal dynamics of the system.

# Abstract: Maternal Inflammation Programs Newborn Immune System

**Published:** 2017-01

**Authors:** Yuval Ginsberg, Nizar Khatib, Zeev Weiner, Ron Beloosesky

**Affiliations:** Department of Obstetrics and Gynecology, Rambam Medical Center, Haifa, Israel

## Introduction

Maternal Gram-negative bacterial infections are common during pregnancy. Attachment of lipopolysaccharide (LPS) to the monocyte Toll-like receptor 4 (TLR4) initiates a signal transduction cascade with activation of nuclear factor kappa B (NFKB) and secretion of inflammatory mediators. We have previously shown that acute maternal exposure to LPS at e18 significantly decreases neonatal cytokine responses to LPS. We sought further to determine whether the offspring cascade of immune response is programmed by maternal inflammation.

## Materials and Methods

Pregnant Sprague-Dawley rats (*n*=4) at 18 days’ gestation received intraperitoneal injections of saline (Control) or LPS (500 μg/kg). Male and female pups were delivered spontaneously (e21) and allowed to mature. At postnatal day 24 (p24), the rats were injected with either LPS (100 mg/kg body weight) or granulocyte-macrophage colony-stimulating factor (GMCSF; 0.2 μg). White blood indices and NFKB activation were determined at baseline and at three hours following i.p. LPS (100 μg/kg) injection. Bone marrow production and secretion of monocytes and granulocytes were determined 3 hours following i.p. GMCSF injection.

## Results

Newborns of LPS-treated dams had significantly higher basal neutrophil and lower lymphocyte percentages than Control newborns, though similar monocyte percentages. Following LPS injection, NFKB activation (phosphorylated P65) was significantly lower in the WBCs of newborn of LPS-treated dams. In response to GMCSF injection, newborns of LPS-treated dams demonstrated higher WBC count (4.6±2.3 vs. 3.2±0.6 * 1,000/μL) and increased monocyte percentages (5.7%±2.2% vs. 3.9%±1.5%) than Control newborns.

## Conclusions

The attenuated serum proinflammatory cytokine response in the offspring following prenatal maternal exposure to LPS is not due to lower serum percentage of cytokine-producing cells (monocytes) or bone marrow reserve monocytes, but rather due to a decrease in sensitivity and in the activation of the NFKB following LPS stimulation.

# Abstract: Immature Myeloid Cells in Reproductive System Versus Tumor Angiogenesis

**Published:** 2017-01

**Authors:** Rivi Hertz, Shahar Kol, Ofer Fainaru

**Affiliations:** 1 Laboratory for Reproductive Sciences, Clinical Research Institute, Rambam Medical Center, Haifa, Israel; 2 IVF Unit, Rambam Medical Center, Haifa, Israel

## Introduction

In close resemblance to other inflammatory states, gonadotropin stimulation and placental development is accompanied by an influx of inflammatory cells, such as neutrophils and monocyte-derived effector cells, into the stroma. Ovarian follicular development, corpora lutea formation, and placental development are examples of the few physiologic events in which the formation of new blood vessels from existing ones occurs, i.e. angiogenesis takes place. Since the unique properties of immature myeloid cells (IMCs) populating these physiologic versus malignant tissues have not been explored, we sought to determine whether ovarian stimulation and placental development leads to an influx of proangiogenic immature myeloid as observed in tumors.

## Materials and Methods

We used 4–5-week-old C57Bl6J-Cx3CR1GFP transgenic mice, in which granulocytic (Ly6G+) and monocytic (Ly6C+) IMCs could be defined by flow cytometry. For global gene expression, IMCs were isolated by flow cytometry, and RNA prepared and analyzed by Affymetrix gene microarrays. Validation of single gene expression was performed by qPCR.

## Results

We detected a significant enrichment (*P*<0.01) of the Ly6Gmed/Ly6Chigh monocytic IMC subpopulation in tumor-derived CD45+ hematopoietic cells compared to placenta, paralleled by a concomitant, more than 2-fold decrease (*P*< 0.01) of the Ly6Ghigh/Ly6Cmed granulocytic IMC subpopulation. In gonadotropin-stimulated ovaries, we observed a ~2.5-fold increase in Ly6Ghigh/Ly6Cmed granulocytic IMCs compared to unstimulated controls. We next assessed the global transcriptional signature of tumor-derived IMCs (T-IMCs) compared to placental IMCs (P-IMCs). Analysis of the top overexpressed genes in T-IMCs revealed several key players in tumor angiogenesis, including Sema3a and matrix metalloproteinases such as Mmp2, Mmp3, Mmp13, and Mmp14.

## Conclusions

IMC subpopulations diverge in tumor versus reproductive tissues, favoring monocytic IMCs in the former, and granulocytic IMCs in the latter. This divergence is associated with unique expression of proangiogenic genes. Selective targeting of these genes may thus be further investigated as selective angiogenic therapies for cancer, placental disease, and ovarian hyperstimulation.

# Abstract: Maternal Inflammation Programs Fetal Renal Development

**Published:** 2017-01

**Authors:** Nizar Khatib, Yuval Ginsberg, Geula Klorin, Edmond Sabo, Zeev Weiner, Ron Beloosesky

**Affiliations:** 1 Department of Obstetrics and Gynecology, Rambam Medical Center, Haifa, Israel; 2 Department of Pathology, Rambam Medical Center, Haifa, Israel

## Introduction

Fetal growth restriction and stress are strongly associated with chronic conditions in later life, including hypertension, diabetes, and metabolic syndrome. Nephrin is an essential protein necessary for the function of the renal filtration barrier. We sought to determine the effect of maternal inflammation on the development and function of the male offspring kidney in early adulthood.

## Materials and Methods

Pregnant Sprague-Dawley rats (*n*=4 in each group) received intraperitoneal injections of LPS (50 μg/kg) or saline injections on days 14, 16, and 18. The pups were then delivered spontaneously. At 3 months of age the offspring were sacrificed; serum was analyzed for blood urea nitrogen (BUN) and creatinine levels, kidneys were harvested for immunofluorescence analysis of nephrin and glomeruli count per area in the renal cortex, and a comparison made between the two groups.

## Results

At birth, the offspring of LPS-treated dams were significantly smaller than the offspring of the saline-treated dams (6±0.7 vs. 6.6±0.4 g, respectively). At 3 months of age offspring weights were similar, but offspring of LPS-treated dams had heavier kidneys compared to male offspring of saline-treated dams (1.59±0.22 vs. 1.34±0.18 g, respectively, *P*<0.05). Relative nephrin fluorescence intensity and nephrin fluorescence per area were reduced by more than 50% in the kidney cortex of male offspring exposed to LPS in pregnancy compared to controls (Figure 1), as were glomerulus counts per area (2.16±0.06 vs. 1.48±0.3, respectively, *P*<0.05). Offspring of both groups had similar serum creatinine and BUN levels.

**Figure 1:** Khatib et al. Nephrin Immuno-fluorescence of Renal Cortex for Normal (top) and LPS (bottom) Groups.

## Conclusion

Sustained maternal inflammation induced intrauterine growth restrictions and a reduction in offspring glomeruli nephrin and glomerulus count per area. No changes in renal function were demonstrated. Longer follow-up is needed to assess long-term function of the kidney and development of kidney-associated diseases in adulthood.

# Abstract: The Effect of Weaning Age on Histological and Morphological Changes in the Small Intestine

**Published:** 2017-01

**Authors:** Yonatan Crispel, Zeev Hochberg, Ranya Khamaise, Edmond Sabo, Ron Shaoul

**Affiliations:** 1 Neurophysiology Unit, Rambam Medical Center, Haifa, Israel; 2 The Ruth & Bruce Rappaport Faculty of Medicine, Technion–Israel Institute of Technology, Haifa, Israel; 3 Pediatric Gastroenterology Institute, Rambam Medical Center, Haifa, Israel; 4 Pathology Institute, Rambam Medical Center, Haifa, Israel

## Introduction

Child development and growth is influenced by many genetic and environmental factors, hence the importance of further studying the issue of weaning and its effects on morphological changes in the intestinal tract.

## Objective

To examine the relationship between weaning age and the morphological changes that occur in rat intestine.

## Materials and Methods

This study was conducted on 25 laboratory rats, 5 rats in each study group. Normally rats are weaned at day 21 of life—these rats served as control group. Early weaning occurs at the age of 16 days; late weaning occurs at 26 days. From each rat we microscopically examined 7 sites along the small intestine with software that enables quantification of the villi and histological intestine changes.

Young rats defined as rats sacrificed at 21 days old, were divided into two groups: one group underwent early weaning at the age of 16 days, while the other group underwent weaning at 21 days (served as control group). Adult rats are defined as rats sacrificed at the age of 90 days; in the adult category another group was added, namely rats that underwent late weaning at 26 days. After weaning at different times, rats were sacrificed and their intestinal tissues preserved in formaldehyde and then stained with H&E stain.

## Results

We found that in the young group of rats those who were weaned early (day 16) had statistically significant lower (*P*<0.005) villus–crypt ratio (villus height:crypt depth) compared to rats weaned on day 21. The thickness of the villus in the group of rats weaned on day 16 was higher versus the group weaned on day 21. In the older group, we found that the villus–crypt ratio in the rats weaned on day 16 was higher compared to rats weaned on day 21 or day 26. The thickness of the villus in the group weaned on day 16 was higher versus the group weaned on day 21 or day 26. No statistical significance was observed for the thickness of the villus between the control group and the group of rats weaned on day 26.

## Conclusions

There was a relationship between the age of weaning and morphological and histological changes in the small intestine. The noted changes may reflect intestinal adaptation to improve nutrients absorption.

# Abstract: Functional Brain Imaging in Critically Ill Children at the Bedside by Combining High-density EEG Recordings, Source Modeling, and Multi-modal Sensory Stimulation

**Published:** 2017-01

**Authors:** Danny Eytan, Elizabeth W. Pang, Sam Doesburg, Vera Nenadovic, Peter Laussen, Anne Marie Guerguerian

**Affiliations:** 1 Department of Critical Care Medicine, The Hospital for Sick Children, Toronto, Canada; 2 Department of Pediatric Critical Care, Rambam Medical Center, Haifa, Israel; 3 Department of Neurology, the Hospital for Sick Children, Toronto, Canada; 4 Behavioural and Cognitive Neuroscience Institute, Vancouver, Canada

## Introduction

Acquired brain injury is a common cause for hospitalization in the pediatric intensive care unit (PICU) associated with an immense personal, social, and economic burden. The goals during treatment in the intensive care are to minimize secondary injury and tailor interventions to optimize outcomes. Timely detection of secondary events or periods of deterioration are critical to allow intervention in order to modify the course of disease. Accordingly, advanced bedside neuromonitoring may offer opportunities for early detection of neurological deterioration and thus lead to improved outcomes.

## Objective

The aim of this study was to develop and test the feasibility of a novel bedside functional imaging set-up aimed at examining cortical brain activation patterns by combining high-density EEG recordings, multi-modal sensory stimulation (auditory, visual, and somatosensory), and EEG source modeling.

## Materials and Methods

This prospective single-center study recorded EEG during multi-modal stimulation in children with severe brain injury (*n*=5) in a pediatric critical care unit, and pediatric healthy controls (*n*=10). Source modeling was performed to test the hypothesis that activation from specific functional brain systems could be obtained at the bedside. Relation to location of injury was evaluated.

## Results

The validation of this system in both healthy and critically ill brain-injured children is demonstrated with modality-specific source-reconstructed cortical activation patterns. By combining stimulation with different modalities, most of the cortical surface can be monitored for changes in functional activation. Application of this set-up in children with anatomically well-defined brain injury suggests an association between these injuries and the activation patterns. Moreover, we demonstrated the use of this set-up to examine higher-level cortical processing with an auditory oddball paradigm involving presentation of the patient’s own name.

## Conclusions

This proof-of-concept study presents a novel tool with the potential to add an additional dimension of information in critically ill children that may aid in guiding treatment, detecting secondary deterioration, and prognosticating outcome.

# Abstract: Children Post Hematopoietic Stem Cell Transplantation (HSCT) admitted to Pediatric Intensive Care Unit (PICU): The Rambam Experience

**Published:** 2017-01

**Authors:** Roni Gefen, Joseph Ben-Ari, Eli Hershman, Iris Porat, Myriam Weyl Ben-Arush, Irina Zaidman

**Affiliations:** 1 Pediatric Bone Marrow Transplant Unit, Ruth Rappaport Children’s Hospital, Rambam Medical Center, Haifa, Israel; 2 Pediatric Intensive Care Unit, Ruth Rappaport Children’s Hospital, Rambam Medical Center, Haifa, Israel

## Introduction

Hematopoietic stem cell transplantation (HSCT) could be the only curative option for children with malignant and non-malignant disorders, but it carries risk of complications, sometimes requiring transfer to pediatric intensive care unit (PICU). Historically, the mortality of these patients was very high.

## Objective

Presentation of retrospective data from pediatric HSCT patients admitted to PICU.

## Materials and Methods

From 2005 to 2015, 213 pediatric HSCTs were performed at Rambam Medical Center (60% allogeneic); with 27 admissions to PICU (12.5% of transplants).

## Results

The admission rate was three times higher in allogeneic transplants vs. autologous. The main indication for PICU admission was respiratory complications, followed by CNS (e.g. coma, seizures), renal, cardiovascular, and other (gastrointestinal, veno-occlusive disease) complications. At transfer, the vast majority of patients (25 out of 27) had failure of only 1 or 2 systems.

The median stay at PICU was 5 days (range: 1–37). Sixteen children (70%) required ventilation; 12 (44%) required inotropes, and 5 (19%) required dialysis. All ventilated patients were allogeneic HSCTs. Overall, 23 out of 27 (85%) deteriorated to multiorgan failure during PICU hospitalization.

The mortality rate during PICU hospitalization was 57%. All post-discharge deaths were secondary to cancer relapse. The PICU survival rate according to number of supported vital systems (respiratory, blood pressure, and kidney) was 87% for children with up to 1 supported system, compared to 10% for those with 2–3 supported systems.

## Conclusions

During the study period, 12.5% of pediatric HSCT patients required PICU assistance. The most common indication for transfer was respiratory problems; indeed, the majority of patients required ventilation. The mortality risk in PICU is above 50%; number of involved vital systems seems to be a prognostic factor. Conversely, transplanted children alive at the end of PICU hospitalization have the potential for long-term survival; none died of HSCT complications. These data encourage prompt involvement of the PICU team early in the course of complications post-HSCT. Further prospective multi-center studies are needed in order to improve outcomes for this high-risk population.

# Abstract: Cleaning and Infection Control of Respiratory Devices Used by CF Patients: A Preliminary Study

**Published:** 2017-01

**Authors:** Eynav Manor, Michal Gur, Yuval Geffen, Lea Bentur

**Affiliations:** 1 Pediatric Pulmonary Institute and CF Center, Ruth Rappaport Children’s Hospital, Rambam Medical Center, Haifa, Israel; 2 Microbiology Laboratory, Rambam Medical Center, Haifa, Israel; 3 The Ruth & Bruce Rappaport Faculty of Medicine, Technion–Israel Institute of Technology, Haifa, Israel

## Background

Respiratory treatment regimen for cystic fibrosis (CF) patients includes respiratory devices. The possibility of contamination of respiratory devices has not been adequately addressed.

## Objective

To determine if respiratory devices are contaminated after use, and if the cleaning guidelines for nebulizers are effective for respiratory devices.

## Methods

Patients brought in their respiratory devices on their routine clinic visit. Swabs were taken from the devices before and after standard cleaning, then sent to be cultured for bacterial count. Total colony-forming units count (CFU) was determined, and predominant colonies were identified using matrix-assisted laser desorption/ionization time-of-flight mass spectrometer (MALDI-TOF-MS) technology.

## Results

Thirty devices were collected from 23 patients. Most of the devices (28/30) were contaminated when brought to the clinic. Complete bacterial eradication was achieved in 15 (50%) and partial eradication in 9 (30%) samples. In four samples the cleaning was ineffective. Median CFU decreased significantly from 1,250 (IQR 25%–75%, 175–10,000) to 0 (IQR 25%–75%, 0–700) before and after cleaning, respectively (*P*<0.0001). The predominant organisms were identified in only 5 samples, and there was no concordance with sputum culture results.

## Conclusions

Respiratory devices are contaminated after use, and appropriate cleaning can reduce contamination. The effect of these contaminated devices on disease progression in CF patients is unclear. There is a need for infection control guidelines for the growing list of respiratory devices.

# Abstract: The Use of Telehealth (WhatsApp and Skype-based Communications) in Patients with Cystic Fibrosis

**Published:** 2017-01

**Authors:** Michal Gur, Vered Nir, Anna Teleshov, Ronen Bar-Yoseph, Eynav Manor, Gizelle Diab, Lea Bentur

**Affiliations:** 1 Pediatric Pulmonary Institute and CF Center, Ruth Rappaport Children’s Hospital, Rambam Medical Center; 2 The Ruth & Bruce Rappaport Faculty of Medicine, Technion–Israel Institute of Technology Haifa, Israel

## Background

Poor communications between cystic fibrosis (CF) patients and health care providers may result in gaps in knowledge and misconceptions about medication usage, which can lead to poor adherence. We aimed to assess the feasibility of using WhatsApp and Skype digital communications to improve patient comprehension and medicating practices.

## Methods

The pilot study included CF patients aged >8 years in a single medical center assigned to two groups: with intervention, and without intervention (control group). Each patient in the intervention group received Skype-based online video chats and WhatsApp messages from members of the multidisciplinary CF team. CF questionnaire, revised (CFQ-R) scores, knowledge, adherence (based on CF My Way questionnaire), and patient’s satisfaction were evaluated before and after a three-month period. Feasibility was assessed by session attendance and an acceptability and satisfaction survey. Descriptive analysis and paired and non-paired *t* tests were employed as applicable.

## Results

Eighteen patients were recruited to the study (9 in each group). Each intervention group participant engaged in 4–6 Skype video chats, and received 22–45 WhatsApp messages. CFQ-R scores, knowledge, adherence, and patient satisfaction responses were similar in both groups before and after the three-month intervention.

## Conclusions

A telehealth-based approach, using Skype video chats and WhatsApp messages, was feasible and acceptable to patients in the pilot study. A larger and longer multi-center study is warranted to examine the efficacy of these interventions in improving knowledge, adherence, and communications.

# Abstract: Genomic Analysis in Hereditary Kidney Disorders: Insights from the Rambam Nephro-genetic Project

**Published:** 2017-01

**Authors:** Daniella Magen, Ayala Ophir, Alina Kurolap, Adi Mory, Hagit Baris-Feldman

**Affiliations:** 1 Institute of Pediatric Nephrology, Rambam Medical Center, Haifa, Israel; 2 Laboratory of Molecular Medicine, Rambam Medical Center, Haifa, Israel; 3 The Ruth and Bruce Rappaport Faculty of Medicine, Technion–Israel Institute of Technology, Haifa, Israel; 4 The Genetics Institute, Rambam Medical Center, Haifa, Israel

## Introduction

Hereditary monogenic kidney disorders account for ~25% of early-onset chronic kidney disease (CKD). Advancement in next-generation sequencing (NGS) technologies has dramatically improved the detection rate of hereditary kidney disorders, enhancing accurate molecular diagnosis, genetic counseling, tailored medical therapy, and careful assessment of potential living-related kidney donors.

## Objective

The Rambam Pediatric Nephrology Institute is a referral center for consanguineous communities from northern Israel and the Palestinian Authority, characterized by a high prevalence of hereditary kidney disorders. As such, we aimed at exploring the molecular basis of suspected monogenic kidney disorders of undiagnosed etiology in all affected children under our care.

## Materials and Methods

After thorough clinical evaluation, all patients aged 0–18 years with suspected hereditary renal disorders, or with CKD/end-stage renal disease (ESRD) of unknown etiology, underwent genetic analysis by either TruSight One (TSO) clinical exome panel, whole-exome sequencing (WES), or targeted candidate-gene sequencing.

## Results

During a two-year period, 35 patients from 28 families were studied; 16 families (57%) were consanguineous, and 20 families (71.4%) presented with chronic renal failure (CRF)/ESRD. TSO, WES, and targeted sequencing were utilized, respectively, in 23 (82.15%), 3 (10.7%), and 2 (7.15%) families. Overall, disease-causing variants were identified in 15 families (53.5%). In 14 families (50%), molecular diagnosis had a significant impact on clinical decision-making and medical management, leading to: changes in suspected diagnosis, withdrawal of immunomodulatory therapy, exclusion of potential living-related kidney donors carrying disease-causing risk variants, establishment of prenatal diagnosis, and offering genetic counseling. Moreover, the phenotypic spectrum and inheritance pattern of two previously identified genetic disorders were unexpectedly expanded, and three novel disease-causing genes were identified using WES.

## Conclusions

Our results indicate a significantly high yield of genetic aberrations in pediatric patients with suspected hereditary kidney disorders, most probably attributed to the high prevalence of consanguinity within the studied population. Our findings provide evidence for the crucial impact of genetic diagnosis on clinical decision-making and on personalized patient care. Taken together, we suggest that genetic analysis should become standard of care in children with suspected hereditary kidney disease or with early-onset CKD/ESRD of unknown etiology, even in the absence of positive family history for genetic kidney disease or consanguinity.

# Abstract: The Coagulation Milieu in Different Organs Is Dynamic and Affects Metastatic Predilection and Growth

**Published:** 2017-01

**Authors:** Neta Nevo, Shourouk Ghanem, Ayelet Ben-Barak, Myriam Ben Arush, Benjamin Brenner, Yona Nadir

**Affiliations:** 1 The Joan and Sanford Weill Pediatric Hematology Oncology and Bone Marrow Transplantation Division, Ruth Rappaport Children’s Hospital, Haifa, Israel; 2 Thrombosis and Hemostasis Unit, Rambam Medical Center, Haifa, Israel; 3 The Ruth & Bruce Rappaport Faculty of Medicine, Technion–Israel Institute of Technology, Haifa, Israel

## Introduction

Metastasis occurs most commonly in the liver, lung, bone, and brain, suggesting preference to specific organs. We hypothesized that organ microcirculation anticoagulant environment could predispose to tumor cell retention and acquirement of a less aggressive phenotype.

## Materials and Methods

Analysis of coagulation factors was performed by immunostaining, ELISA, and heparanase procoagulant activity assay.

## Results

In normal mice, the expression of heparanase, tissue factor (TF), tissue factor pathway inhibitor (TFPI), and TFPI-2 was low in the microcirculation of the liver, lung, bone, brain cortex, and kidney cortex, and high in the microcirculation of the brain sub-cortex, bone marrow, kidney medulla, subcutis, and in smooth and striated muscles. B16 (F10) melanoma cells, injected subcutaneously to C57BL/6 mice, were found in the blood vessels of metastasis with lower levels of heparanase, TF, TFPI, and TFPI-2 compared to those of the primary tumor (*P*<0.01). In these metastatic mice, liver and lung microcirculation turned to present high levels of heparanase, TF, TFPI, and TFPI-2. Human umbilical endothelial cells incubated with B16 melanoma cells medium expressed increased levels of heparanase, TF, TFPI, and TFPI-2, and the effect was reversed by the heparanase procoagulant activity inhibitory peptide (*P*<0.01). The addition of that peptide to cell cultures of U87 human glioma and MDA-231 human breast carcinoma significantly decreased levels of heparanase in the medium (*P*<0.001). Other anticoagulant drugs, including enoxaparin, dabigatran, and rivaroxaban, did not exert these effects on endothelial or tumor cells. Results were also confirmed in stained tissue samples of two children suffering from neuroblastoma, demonstrating dynamic levels of heparanase during their illness.

## Conclusions

Metastasis has predilection to organs with low levels of heparanase, TF, TFPI, and TFPI-2, where tumor cells are preserved. Tumor affects the organ microcirculation phenotype and converts it to a procoagulant microcirculation that enables tumor cell growth.

# Abstract: The Essential Role of PAR1 Signaling in Clinical G-CSF Induced HSPC Mobilization and Its Inverse Regulation by Murine Hematopoietic and Stromal Cells

**Published:** 2017-01

**Authors:** Neta Nevo, Shiri Gur-Cohen, Orit Kollet, Tsila Zuckerman, Benjamin Brenner, Yona Nadir, Irina Zaidman, Myriam Ben Arush, Tsvee Lapidot

**Affiliations:** 1 Pediatric Hemato-oncology, Rambam Medical Center, Haifa, Israel; 2 Department of Immunology, Weizmann Institute of Science, Rehovot, Israel; 3 Hematology and Bone Marrow Transplantation Division, Rambam Medical Center, Haifa, Israel

## Introduction

Hematopoietic stem and progenitor cell (HSPC) egress and mobilization from the bone marrow are tightly regulated processes. Recently we discovered that factors, traditionally viewed as coagulation- and inflammation-related, also independently regulate mouse bone-marrow long-term hematopoietic stem cells (LT-HSC) repopulating via distinct protease-activated receptor 1 (PAR1) signaling pathways via control of nitric oxide (NO) generation. Herein we aim to decipher whether PAR1 surface expression levels can serve as a predictive factor for mobilization efficiency of human CD34^+^ HSPC.

## Materials and Methods

Peripheral blood samples were obtained from 20 healthy bone marrow donors before and after treatment with granulocyte colony stimulating factor (G-CSF). Mononuclear cells were isolated, stained, and analyzed by fluorescence activated cell sorting (FACS). In addition, we utilized a functional preclinical mouse model.

## Results

PAR1 expression on circulating leukocytes before G-CSF administration positively correlated with higher yields of mobilized leukocytes (*P*<0.001). Furthermore, the percentage of PAR1-expressing CD34+ HSPCs in the blood and their absolute numbers before mobilization positively correlated with peripheral blood leukocyte counts (*P*<0.001) and CD34+ HSPC yield (*P*=0.002). Importantly, we found accelerated neutrophil generation in patients transplanted with mobilized cells expressing higher surface PAR1 levels at baseline (*P*=0.074) and a positive trend in platelet production. We found that PAR1 induces NO generation which is essential for murine G-CSF-induced HSPC mobilization. Co-administration of G-CSF with PAR1 antagonist prevented NO generation, leading to abrogated HSPC mobilization, concomitantly with increased numbers of bone-forming mesenchymal progenitors and bone marrow (BM) retained HSPC. Finally, we report an inverse PAR1 expression and regulation by BM HSPC and mesenchymal stem and progenitor cells (MSPC). G-CSF induced elevation of PAR1 expression on BM HSPCs, enhancing their migration, proliferation, differentiation, and recruitment to the circulation. In contrast, the levels of PAR1 expression were reduced on MSPCs in response to G-CSF treatment, leading to accumulated numbers of immature mesenchymal and osteoblast progenitor cells.

## Conclusions

Our study identifies PAR1 as an essential regulator of human and murine G-CSF induced HSPC mobilization via NO generation and highlights inverse PAR1 signaling as an essential regulator of G-CSF induced HSPC mobilization and MSPC development.

# Abstract: The Utility of Bronchoscopy and Bronchoalveolar Lavage in the Diagnosis of Pulmonary Infections in Immunocompromised Pediatric Patients

**Published:** 2017-01

**Authors:** Suha Rizik, Imad Kassis, Nira Arad-Cohen, Myriam Ben-Arush, Anna Teleshov, Lea Bentur, Fahed Hakim

**Affiliations:** 1 Pediatric Pulmonary Institute and CF Center, Ruth Rappaport Children’s Hospital, Rambam Medical Center, Haifa, Israel; 2 Pediatric Infectious Disease Unit, Ruth Rappaport Children’s Hospital, Rambam Medical Center, Haifa, Israel; 3 Pediatric Hemato-Oncology Division, Ruth Rappaport Children’s Hospital, Rambam Medical Center, Haifa, Israel

## Background

Immunocompromised pediatric patients are at high risk of developing pulmonary infections with high rates of morbidity and mortality. The diversity of possible infectious etiologies, and the possibility of co-infections, makes it difficult to reach a definite infectious diagnosis. These patients are at risk of rapid deterioration, which drives physicians to initiate empiric broad-spectrum antimicrobial therapy while running a wide series of tests to identify specific pathogens, including fiberoptic bronchoscopy (FOB) with bronchoalveolar lavage (BAL).

## Objectives

As microbiological techniques continue to advance in the last decade, we aimed to evaluate the microbial diagnostic rate, and the safety and rate of treatment change based on FOB and BAL findings.

## Methods

We retrospectively reviewed the records of 134 immunocompromised pediatric patients who underwent FOB with BAL during 2006–2014.

## Results

Empiric antimicrobial treatment was initiated in 78% of cases. BAL yielded a definite diagnosis in 47% of patients. Medical management was subsequently changed in 63.2% of patients as a consequence of either a positive or a negative BAL result. No severe complications associated with the BAL procedure occurred.

## Conclusions

In the setting of immunocompromised pediatric patients with suspected pulmonary infections, evaluation using FOB and BAL is a safe and useful diagnostic tool. With both positive and negative results contributing to patient management, this procedure should be considered as an initial diagnostic tool in evaluating immunocompromised pediatric patients with pulmonary manifestations.

# Abstract: Stone Burden and Risk for Surgery Among Children Presenting with Hypercalciuria, Hypocitraturia, and Cystinuria as Underlying Metabolic Causes of Urolithiasis

**Published:** 2017-01

**Authors:** Fadi Zu’bi, Martin Sidler, Elizabeth Harvey, Asal Hojjat, Naimet Naoum, Roberto I. Lopes, Martha Pokarowski, Walid Farhat, Frank Papanikolaou, Joana Dos Santos

**Affiliations:** 1 Department of Surgery, Division of Urology, The Hospital for Sick Children, Toronto, Canada; 2 Division of Nephrology, The Hospital for Sick Children, Toronto, Canada

## Background

Hypercalciuria, hypocitraturia, and cystinuria are the most common underlying stone metabolic abnormalities and account for more than 80% of all renal stones in children. This study compares the impact of those underlying metabolic conditions on stone growth patterns and stone burden in the pediatric population.

## Materials and Methods

We retrospectively reviewed the charts of children with renal stones between 2000 and 2015. Data captured included: imaging (ultrasounds and abdominal X-rays); stone size, number, and location; metabolic workup (24-hour and/or spot urine collection); new stone formation; and stone-related surgical procedures. Differences among metabolic groups were determined using ANOVA. Independent samples *t* test was used to compare scale variables, and we applied the chi-square test for categorical variables. Logistic regression was performed to assess the effect of various parameters on the likelihood of needing surgery.

## Results

During the study period 356 children were seen at a children’s hospital. Metabolic abnormality was identified in 154 (55%) pediatric patients, with the most common being hypercalciuria (*n*=86; 30.7%), hypocitraturia (*n*=20; 7.1%), and cystinuria (*n*=24; 8.6%); mixed and others abnormalities were found in 24 (8.6%) pediatric patients. Hypercalciuria was significantly more prevalent in the younger age groups under 10 years. Cystinuria, on the other hand, was significantly more prevalent in 11–18-year-old patients. Mean stone size at baseline was significantly larger in children with cystinuria compared to those with hypercalciuria (11.2 vs. 7.4 mm, *P*= 0.038), hypocitraturia (11.2 vs. 7 mm, *P*=0.027), and normal metabolic group (11.2 vs. 7 mm, *P*=0.003); a total of 123 (34.5%) patients had to undergo surgery. Median time from first clinic visit to surgery was 102 days (IQR 34–330 days). Stone growth rates were not statistically significant among metabolic groups. Larger stones, cystinuria, and proximal ureteral stones were significantly more likely to necessitate surgical treatment.

## Conclusions

Our data showed no difference in stone growth rate between the three metabolic abnormalities investigated. Stones in children with cystinuria were significantly larger than in other metabolic groups. Initial stone size, proximal ureteral stone, and cystinuria are significant risk factors that predict the need for intervention.

# Abstract: The Impact of the State of Hydration on the Degree of Hydronephrosis in Children

**Published:** 2017-01

**Authors:** Fadi Zu’bi, Joana Dos Santos, Roberto I. Lopes, Asal Hojjat, Abby Varghese, Martin Koyle, Darius Bagli

**Affiliations:** Department of Surgery, Division of Urology, The Hospital for Sick Children, Toronto, Canada

## Background

Hydronephrosis (HN) is an imaging state affected by multiple factors, including hydrodynamic inflow (e.g. hydration), hydrodynamic outflow (e.g. obstruction), and renal tissue compliance (e.g. elasticity). Little attention is usually given to the impact of fluid intake and physiologic diuresis on the dilation of the urinary tract. We propose a protocol to control outflow resistance and provoke maximal physiologic hydration before ultrasonographic imaging for HN.

## Objective

We report preliminary data from an ongoing study which seeks to determine the degree of HN seen on renal ultrasound (US) is impacted by the state of hydration at the time of the study.

## Materials and Methods

This is a prospective study, conducted over 8 months, of renal and bladder of renal and bladder ultrasounds of children with confirmed postnatal HN from birth up to 18 years of age. Children with ureteral dilatation, neurogenic bladder, and lower urinary tract obstruction were excluded. Twenty children were initially recruited and enrolled. Children’s weight pre- and post-hydration have been recorded. After the first post-void renal US (pre-hydration) was done, patients received oral hydration with 15–20 mL/kg water. Renal and bladder US were repeated post-void (post-hydration) 30–60 minutes later. Measured parameters in both the pre- and post-hydration US included anterior-posterior (AP) diameter of renal pelvis, extra-renal pelvis diameter, bladder status during US (full vs. empty), and post-void residual. Our focus was on the change in HN with hydration represented by change in AP diameter (Wilcoxon paired, two-tailed *t* test; *P*<0.05).

## Results

Twenty children were initially recruited and enrolled. Mean age in months was 74.9±50.1 (8.1–165.4), female to male ratio 1:6, 45% with left-sided HN, 25% with right-sided HN, 15% bilateral, and 5% were excluded from analysis. Pre-hydration mean AP diameter was 7.5 mm (IQR 4.8–11). Post-hydration mean AP diameter was 9.6 mm (IQR 7.3–14) with a mean increase of 28% following hydration (*P*<0.001). Kidneys were categorized by percent change in AP diameter (pre- vs. post-hydration); 55% (*n*=11) of kidneys experienced an increase of over 25%.

## Conclusion

The degree of hydronephrosis is significantly impacted by hydration status at the time of the US study. We suggest that a standard protocol of hydration should be considered when sonograhically assessing renal dilatation.

# Abstract: Burns Out, Pneumonia In: Pneumonia Risk in Urgently Intubated Burn Patients. Why, Where and When?

**Published:** 2017-01

**Authors:** Ori S. Duek, Yeela Borenstein, Ben Naftali, Yaron Bar-Lavie, Yehuda Ullmann

**Affiliations:** 1 Department of Plastic & Reconstructive Surgery, Rambam Medical Center, Haifa, Israel; 2 Intensive Care Unit, Rambam Medical Center, Haifa, Israel

## Introduction

Pneumonia is a major cause of morbidity and mortality in burn patients with inhalation injuries. An increased risk of pneumonia has previously been demonstrated in trauma and burn patients urgently intubated in the field or emergency department (ED) settings.

## Objectives

This study investigated the impact of intubation location in burn patients on subsequent development of pneumonia, and evaluated the necessity of urgent intubation.

## Materials and Methods

A retrospective review of medical records for all intubated patients suffering from thermal or trauma injuries (for comparison), admitted to the intensive care unit (ICU) of a single burn center and level I trauma center. Patients undergoing field or ED intubation during the 15-year period ending July 2016 were identified and their records reviewed. Patient demographics, burn mechanism, presence of inhalation injury, location at the time of intubation, presence of associated injury, percentage total body surface area (TBSA) of burn, hospital and intensive care unit length of stay, hospital day of pneumonia diagnosis, and microbiology results were analyzed.

## Results

The study included 102 burn patients and 73 multitrauma (control group) patients. Approximately 63% of patients had airways established in the ED (65 burn patients, 46 control patients), while 37% were intubated in the field. Most of the burn patients were males (80%), with a similar average age in both burn groups (around 41 years) and no major differences in co-morbidities. In the field intubation group, more patients had large burns (45% had TBSA >50%, compared to 29% in the ED intubation group), and fewer patients suffered from small burns (TBSA <20%, 23% field vs. 36% ED). Pneumonia developed during hospitalization in 46% of ED-intubated patients (same percentage for burns and control groups), compared to 81% of patients in the field-intubated control group and 62% in the field-intubated burn group (*P*<0.05). In the control group, 59% of field-intubated patients developed pneumonia after 1 week, compared to 35% of ED-intubated patients (*P*<0.05).

## Conclusions

Field intubation (compared to ED intubation) is a risk factor for the development of pneumonia in burn or multitrauma patients; it must be applied with caution and only when airway patency is at immediate risk.

# Abstract: Trend and Demographic Characteristics of Maxillofacial Fractures in the Populations of Northern Israel in a Level I Trauma Center (1,097 Cases)

**Published:** 2017-01

**Authors:** Omri Emodi, Amir Wolff, Hanna Srouji, Hany Bahouth, Dani Noy, Abu El Naaj Imad, Adi Rachmiel

**Affiliations:** 1 The Department of Oral and Maxillofacial Surgery, Rambam Medical Center, Haifa, Israel; 2 The Ruth & Bruce Rappaport Faculty of Medicine, Technion–Israel Institute of Technology, Haifa, Israel; 3 Trauma Center & Emergency Surgery, Department of General Surgery, Rambam Medical Center, Haifa, Israel; 4 Department of Oral and Maxillofacial Surgery, Baruch Padeh Medical Center, Affiliated with the Faculty of Medicine of Bar-Ilan University, Poriya, Israel

## Introduction

To analyze the pattern and treatment of craniomaxillofacial (CMF) injuries within a Jewish majority and large Arab minority population, in northern Israel.

## Materials and Methods

A 5-year retrospective study evaluated medical records of 1,097 patients treated for CMF fractures. Fracture cause, type, site, and patient demographics were evaluated.

## Results

Patient age ranged from 1 to 94 with an average age of 36.7 years; 52% of the victims were Jews and 48% Arabs. Males were in the majority in both sectors (78.3% vs. 21.7%). The main site of injury was the zygomatic bone (33.5%), followed by nasal bone, orbital, mandible, frontal sinus, and maxillary fractures. The main etiology of injuries was falls (45.4%), with significantly more falls reported by females (52.1% vs. 43.2% in males). Motor vehicle accidents caused injuries more frequently in males. Arabs experienced CMF fractures at a younger age compared to Jews (27.8 and 44.8 average age, respectively). In the elderly, the trend reversed, with Jews being more prone to CMF fractures.

## Conclusions

Compared to their proportion in the population, the Arab sector experiences more CMF injuries. The Jewish elderly population tends to reside in nursing homes where they are more susceptible to accidental falls, while young Arab males are more exposed to motor vehicle accidents and interpersonal violence. Falls were the main cause of injuries particularly in women. This may reflect the women’s fear of reporting domestic violence. We believe that increased government investments in infrastructures and education will lower the incidence of CMF trauma and balance the gap between both sectors and genders.

# Abstract: The Use of Anterior Maxillary Sinus Bone Grafts in Treatment of Orbital Floor Fractures

**Published:** 2017-01

**Authors:** Omri Emodi, Saleh Nseir, Dekel Shilo, Hanna Sroujia, Adi Rachmiel

**Affiliations:** 1 Department of Oral and Maxillofacial Surgery, Rambam Medical Center, Haifa, Israel; 2 Ruth & Bruce Rappaport Faculty of Medicine at the Technion–Israel Institute of Technology, Haifa, Israel

## Introduction

Isolated orbital wall fractures account for 4%–16% of all facial fractures. Even a modest change in the position of the bony walls can have a significant impact on orbital volume and globe position. Alloplastic materials or autogenous bone grafts such as the antral maxillary wall can be used to reconstruct small to medium-sized orbital fractures. The main advantage of an antral wall graft is the intra-oral approach with minimal morbidity.

## Materials and Methods

Nine patients underwent repair of orbital floor fractures using the extra-oral and the intra-oral antral wall approach, seven of them with maxillary antral bone grafts. The patients underwent preoperative CT imaging and a minimum of one year of follow-up.

## Results

The size of the defects ranged from 0.5 to 1.3 cm. Four of the patients experienced minor postoperative complications, i.e. exophthalmos, diplopia, and infraorbital hypoesthesia.

Postoperatively, none of our patients had ocular movement restrictions or complications regarding the maxillary antral wall approach.

## Conclusions

The use of harvested bone grafts from the anterolateral wall of the maxillary sinus is a promising approach for the reconstruction of small to midsize orbital floor defects with minimal complications and excellent cosmetic and functional results.

# Abstract: The Clinical Characteristics and Treatment Outcomes of Condylar Hyperplasia Patients

**Published:** 2017-01

**Authors:** Michal Even-Almos, Amir Totry, Omri Emodi, Adi Rachmiel

**Affiliations:** 1 The Ruth & Bruce Rappaport Faculty of Medicine, Technion–Israel Institute of Technology, Haifa, Israel; 2 Department of Oral and Maxillofacial Surgery, Rambam Medical Center, Haifa, Israel

## Introduction

The aim of this study is to present our clinical experience with patients diagnosed with condylar hyperplasia (CH), describing the clinical characteristics and the esthetic and functional results after treatment by high condylar shave during the active phase.

## Material and Methods

In this retrospective study, 19 patients with condylar hyperplasia (14 females and 5 males, 11–26 years old) were treated and evaluated. All patients underwent a clinical and a radiographic evaluation that included recording their history regarding the onset of the asymmetry, rate of progression, panoramic and anteroposterior (AP) cephalometric X-rays, and SPECT.

The treatment plan for all patients was divided into two stages. The first stage was high condylar shave via Al-Kayat Bramley incision during the active phase of the condylar overgrowth, and the second stage was orthognatic surgery as needed.

Clinical examinations during the follow-up period included assessment of the mouth opening in terms of magnitude and symmetry, observation of facial symmetry, and recording of complications such as facial nerve paralysis.

## Results

Facial symmetry and normal occlusion were achieved by the first procedure in 18 patients, and no further surgeries were needed. One patient underwent a second surgery that included a combination of bilateral or unilateral mandibular osteotomies with or without genioplasty and LeFort 1 maxillary osteotomy to treat the asymmetry.

There were no serious postoperative complications. The frontal branch of the facial nerve was temporarily affected in one case, which was resolved in 2–4 months.

## Conclusions

Mandibular condylar hyperplasia is a condition that causes overgrowth of the mandibular condyle, leading to facial asymmetry, occlusal disturbance, and joint dysfunction. The asymmetry progresses as long as the condylar growth is active.

According to our experience, in order to allow a balanced facial development, we recommend dividing the treatment into 2 stages; the first stage includes suppression of the condylar growth process during the active phase by a high condylar shave procedure. Follow-up and symmetry re-evaluation after puberty at the end of active growth phase should be conducted, in order to consider an orthognathic surgery to restore facial symmetry.

# Abstract: Effects of Extracorporeal Shock Wave Therapy on Distraction Osteogenesis in the Rat Mandible

**Published:** 2017-01

**Authors:** Jiriys George Ginini, Omri Emodi, Gila Maor, Dror Aizenbud, Adi Rachmiel

**Affiliations:** 1 Ruth & Bruce Rappaport Faculty of Medicine at the Technion–Israel Institute of Technology, Haifa, Israel; 2 Department of Oral and Maxillofacial Surgery, Rambam Medical Center, Haifa, Israel

## Introduction

Distraction osteogenesis (DO) has gained widespread clinical use in both orthopedics and craniofacial fields for the treatment of congenital and acquired deformities. However, the long consolidation period and the potential of complication remain major limitations.

The aims of the study are to evaluate the influence of extracorporeal shock wave therapy (ESWT) on DO in rats’ mandible and to determine in which period of the DO the EWST is the most effective to accelerate bone regeneration.

## Materials and Methods

Twenty-four male Sprague-Dawley rats were subjected to DO of the right mandible (latency period, 3 days; distraction period, 10 days at a rate of 0.5 mm/day). Rats were divided to three groups: group I (control) without ESWT, group II received ESWT (0.18 mJ/mm^2^) at latency period, and group III received ESWT (0.18 mJ/mm^2^) at consolidation period.

All animals were sacrificed after 4 weeks of consolidation period, and explants were removed for radiographic, histological, micro-CT, and immunohistochemical (IHC) evaluation.

## Results

Groups II and III showed higher radiopacity compared to group I in the distraction gap. Histological analysis detected more bone formation in the treated groups and thicker newly formed trabeculae in group III compared to the other groups. IHC staining demonstrated significantly increased expression of bone morphogenetic protein 2 (BMP2), vascular endothelial growth factor (VEGF), and osteocalcin in group III compared to the control group (*P*<0.05). Micro-CT showed a significantly higher level of bone mineral density, bone trabecular fraction, and trabecular thickness in group III compared to control group (*P*<0.05).

## Conclusions

ESWT may enhance bone generation and is most effective during the consolidation period.

# Abstract: Short Sample of Heart Rate Variability in Airway Obstruction Model

**Published:** 2017-01

**Authors:** Amit Lehavi, Neta Golomb, Ronit Leiba, Yeshayahu (Shai) Katz, Aeyal Raz

**Affiliations:** 1 Department of Anesthesiology, Rambam Medical Center, Haifa, Israel; 2 Department of Epidemiology, Rambam Medical Center, Haifa, Israel; 3 Department of Anesthesiology, University of Wisconsin, Madison, Wisconsin, USA

## Introduction

Heart rate variability (HRV) is a useful tool for evaluating cardiac and autonomic nervous system activity. It is affected both by the respiratory system and changes in intrathoracic pressure. HRV is usually evaluated over a prolonged time period. Multiple time and frequency domain analyses are used to evaluate changes in HRV. We studied rapid HRV changes during airway obstruction.

## Materials and Methods

Following Institutional Review Board (IRB) approval and signing an informed consent, 40 healthy volunteers underwent a one-minute trial of breathing through a simulated obstructed airway using an 18 cm long, 4 mm internal diameter endotracheal tube. Heart rate was recorded during three sets of one minute of control normal breathing, followed by one minute of obstructed breathing. Time and frequency domain analysis of heart rate was performed to compare HRV during normal versus obstructed breathing.

## Results

During obstructed breathing, time elapsing between two consecutive R waves on the electrocardiogram (R-R interval) decreased from 893 to 827 milliseconds (ms). Time domain variables dramatically increased: R-R interval standard deviation increased from 65 to 108 ms (*P*<0.0001), root mean square of successive differences increased from 61 to 82 ms (*P*=0.001), number of pairs of successive R-R intervals that differed by more than 50 ms (NN50) increased from 16.5 to 25.3 events (*P*<0.0001), and the proportion of NN50 divided by total number of R-R intervals increased from 26.6% to 35.1% (*P*= 0.001). Frequency domain variables demonstrated a marked (more than 4-fold) increase in the low-frequency power (*P*<0.0001), but no significant changes were found in the high-frequency power.

## Conclusions

We noticed a rapid and robust increase in HRV during periods of obstructed breathing. The change was significant enough to be detected even during a short, one-minute sampling window. It was obvious in both frequency, time domain, and frequency analyses. These findings suggest that HRV may be useful for rapid detection of critical events such as airway obstruction during anesthesia. The usefulness of this technique during anesthesia should be further verified before widespread application.

# Abstract: Hypothyroidism, Autoimmunity and Systemic Characteristics of Salivary Tumors

**Published:** 2017-01

**Authors:** Yair Israel, Adi Rachmiel, Gil Ziv, Rafael Nagler

**Affiliations:** 1 Department of Oral and Maxillofacial Surgery, Rambam Medical Center, Haifa, Israel; 2 The Ruth and Bruce Rappaport Faculty of Medicine, Technion–Israel Institute of Technology; 3 Department of Otolaryngology Head and Neck Surgery, Rambam Medical Center, Haifa, Israel; 4 Rappaport Family Institute for Research in the Medical Sciences, Technion–Israel Institute of Technology, Haifa, Israel

## Introduction

We examined demographic, ethnic, and systemic characteristics of patients diagnosed with salivary benign and malignant tumors in order better to understand their possible etiological roles.

## Materials and Methods

Medical records of 287 patients who had received definitive treatment for primary salivary glands tumors in Rambam Medical Center, Haifa, Israel, were examined.

## Results

Of 287 cases, 214 had benign tumors and 73 malignant tumors. Mean age at diagnosis in the malignant group was 56.4 years (median 57.0) and 48.5 years (median 51.0) in the benign group, a highly significant difference (*P*=0.001). Females represented 45% of patients in the malignant group and 59% in the benign group (*P*=0.037). The baseline variables of ethnic origin, alcohol consumption, and cigarette smoking rates were not significantly different (*P*>0.05) between the groups. While the prevalence rates of patients with diabetes mellitus (DM), cerebro-vascular attack (CVA) and/or ischemic heart disease (IHD), hypertension (HTN), and hyperlipidemia (HL) were not significantly different between the two groups (*P*>0.05), the percentage of patients with hypothyroidism (10.2%) in the benign group was significantly higher than in the malignant group (1.4%) (*P*=0.021).

## Conclusions

The results are discussed with respect to the available literature and support the hypothesis that autoimmunity may play a significant etiological role in benign but not malignant salivary tumors, further supported by the significantly higher prevalence of women in the benign group.

# Abstract: Interobserver and Intraobserver Agreement for Letournel Acetabular Fracture Classification System Using Three-dimensional Models

**Published:** 2017-01

**Authors:** Doron Keshet, Eli Peled, Doron Norman, Yaniv Keren

**Affiliations:** Department of Orthopedic Surgery, Rambam Medical Center, Haifa, Israel

## Introduction

The complex Judet–Letournel classification system of acetabular fractures is most commonly used by orthopedic trauma surgeons. Three-dimensional printing technology has become available in recent years and is used as a diagnostic and planning tool for executing surgeries. The aim of this study was to evaluate the reliability of the classification system of Letournel using CT imaging and 3-D models. In addition, we examined the contribution of 3-D printed models in diagnosis, classification, and subsequently the surgical approach chosen.

## Materials and Methods

We examined 7 cases of acetabular fractures; for each case a file was prepared including CT imaging and an accurate 3-D printed model. A total of 18 orthopedic trauma surgeons responded to questionnaires, regarding the individual fracture classification based on Letournel as well as their preferred surgical approach. The rates of agreement were analyzed among participant responses regarding classification and surgical approach; these were the rates of inter- and intra-observer agreement.

## Results

The inter-observer agreement regarding fracture classification based on CT imaging was moderate (Kappa=0.438). The inter-observer agreement based on the 3-D models was higher, but still moderate (Kappa=0.553; *P*<0.001). It was also found that the degree of inter-observer agreement for the preferred surgical approach according to CT was fair (Kappa=0.342), and that the degree of inter-observer agreement according to the 3-D printed models was lower, but still fair: Kappa=0.287 (*P*=0.005). The rate of intra-observer agreement for the fracture classification, using both of the imaging modalities, was found to be moderate (Kappa=0.476). The rate of intra-observer agreement regarding the preferred surgical approach when using both of the imaging modalities was also moderate (Kappa=0.407).

## Conclusions

The data suggest only moderate rates of agreement within the Letournel classification system for acetabular fractures. Using a 3-D printed model increases the rates of agreement regarding the fracture classification but lowers the rates of agreement regarding the preferred surgical approach. Three-dimensional models allow for better spatial understanding and enable more accurate fracture classification. It is possible that the ability of the surgeon to hold a 3-D model in their hands as part of the surgical planning could contribute to better understanding of the fracture.

# Abstract: Trends in Authorship of Papers in Major Ophthalmology Journals by Gender, 2002–2014

**Published:** 2017-01

**Authors:** Michael Mimouni, Shiri Zayit-Soudry, Ori Segal, Yoreh Barak, Arie Y. Nemet, Shiri Shulman, Noa Geffen

**Affiliations:** 1 Department of Ophthalmology, Rambam Medical Center, Haifa, Israel; 2 Department of Ophthalmology, Meir Medical Center, Haifa, Israel; 3 Department of Ophthalmology, Tel Aviv Medical Center, Tel Aviv, Israel

## Introduction

The purpose of this study was to evaluate trends in the prevalence of women authors in ophthalmology over recent years.

## Materials and Methods

Using the PubMed search engine, we conducted an observational study of trends in gender distribution of all authors in 6 leading ophthalmology journals during January 2002 to December 2014. In multi-authored papers, the first listed author is often the lead investigator and the last the senior author. Therefore, the full names and authorship positions (first, middle, or last) of all authors in every article were collected. A Google-based name identifier was used to assign the gender of authors.

## Results

A total of 102,254 authors from 23,026 published articles were analyzed. There was a significant rise over time in the percentage of women authors, with a steeper slope for first authors than for last (*P*<0.001). The rise in the percentage of women authors was similar in basic and clinical research but was steeper for first authorship than for last authorship (*P*<0.001). In all three authorship positions (first, middle, and last), women’s contributions were consistently higher in basic research publications. The rise in the percentage of women authors was significantly steeper for general journals than for subspecialty journals (*P*<0.001). There was no significant rise for last authorship in subspecialty journals.

## Conclusions

Despite an overall increase in the contribution of women to the field of ophthalmology, contributions to papers published in subspecialty ophthalmology journals and the proportion of women listed as last authors remain low.

# Abstract: Cycloplegic Autorefraction in Young Adults: Is It Mandatory?

**Published:** 2017-01

**Authors:** Michael Mimouni, Lilach Zoller, Josefa Horowitz, Tamara Wygnanski-Jaffe, Yair Morad, Eedy Mezer

**Affiliations:** 1 Department of Ophthalmology, Rambam Medical Center, Haifa, Israel; 2 Maccabi Health Services, Haifa, Israel; 3 Carmel Medical Center, Haifa, Israel; 4 Goldschlager Eye Institute, Shiba Medical Center, Tel-Hashomer, Israel; 5 Sackler Faculty of Medicine, Tel Aviv University, Tel Aviv, Israel; 6 Assaf-Harofeh Medical Center, Tzrifin, Israel; 7 The Ruth & Bruce Rappaport Faculty of Medicine, Technion–Israel Institute of Technology, Haifa, Israel

## Introduction

The precise correction of refractive error is especially important in young adults. It is unclear whether or not cycloplegic refraction is necessary in this age group. The purpose of this study was to compare the non-cycloplegic and cycloplegic spherical equivalent (SE) refractive error measured in young adults.

## Materials and Methods

This was a prospective study of 1,400 eyes (*n*=700) of enlisted soldiers aged 18–21 years who were consecutively evaluated in an outpatient army ophthalmology clinic. A drop of cyclopentolate 1% was installed twice 10 minutes apart, and cycloplegic refraction was performed in both eyes 40 minutes later using an auto-refractor. The difference between non-cycloplegic and cycloplegic refractive measurements was analyzed.

## Results

The mean difference in SE between non-cycloplegic and cycloplegic measurements was 0.68±0.83 D (95% CI 0.64–0.72). Significantly greater differences were observed in hypermetropes than myopes (1.30±0.90 D versus 0.46± 0.68 D, *P*<0.001). Moderate hypermetropes (2 to 5 D) demonstrated significantly greater refractive error than mild (0.5 to 2 D) or severe (>5 D) hypermetropes (1.71±1.18 D versus 1.19±0.74 D and 1.16±1.08 D, respectively, *P*<0.001).

## Conclusions

Young hypermetropic adults possessed +1 to +2 D of latent hypermetropia. In contrast, young myopic adults revealed pseudomyopia of −0.5 D. Cycloplegic refraction should be performed in young hypermetropic adults complaining of various signs of asthenopia.

# Abstract: A Method for the Selection of Cataract Disintegrating Compounds and Their Use for Reversal of Crystalline Lens Opacification

**Published:** 2017-01

**Authors:** Michael Mimouni, Marina Chemerovski-Glikman, Yarden Dagan, Esraa Haj, Lihi Adler-Abramovich, Daniel Segal, Ehud Gazit, Shiri Zayit-Soudry

**Affiliations:** 1 Department of Ophthalmology, Rambam Medical Center, Haifa, Israel; 2 Department of Molecular Microbiology and Biotechnology, George S. Wise Faculty of Life Sciences, Tel Aviv University, Tel Aviv, Israel

## Introduction

We describe a novel *ex vivo* assay for examining the efficacy of various compounds for the reversal of human crystalline lens opacification in the search for pharmacological treatment for cataract.

## Materials and Methods

After obtaining informed consent, fragments of cataractous lenses were collected from patients undergoing routine clear corneal incision phacoemulsification cataract surgery. *Ex vivo* cataract samples were then incubated with several concentrations of various compounds being screened for cataract-modulating effect. Reversal of protein aggregates was monitored several times a day for up to 48 h, using the common turbidity assay. Samples were stored at 37°C with constant shaking between measurements.

## Results

Each compound selected for screening was tested on cataract samples obtained in triplicates from 5–10 different patients. Initial total protein concentrations were in the range of 1–10 mg/mL, and, after adjustment for optimal absorbance, final concentrations were in the range of 0.5–3 mg/mL. Of the 15 compounds screened, 5 showed no effect on protein turbidity measurement over time, while 5 showed inconsistent results. Treatment with five compounds resulted in significant reduction in the optical density of the solution, reflecting restoration of transparency of the cataractous samples, two of which showed a dose-dependent effect. Amongst them, each agent demonstrated different efficacy and unique kinetics.

## Conclusions

This simple yet innovative experimental approach implements spectrophotometric principles to enable direct testing of the impact of potential compounds on disintegration of actual human crystalline lens material *ex vivo*. Further biophysical studies, currently conducted by our group, will provide mechanistic insight into the cataract reversal effect.

# Abstract: Comparison of Single-Vessel Coronary Bypass Grafting, Minimally Invasive, Direct Coronary Artery Bypass (MIDCAB) Versus Off-Pump Coronary Surgery by Sternotomy (OPCAB)

**Published:** 2017-01

**Authors:** Zvi Peled, Oved Cohen, Ariel Rougin, Keren Bitton Worms, Victor Kerzman, Gil Bolotin

**Affiliations:** 1 Department of Cardiac Surgery, Rambam Medical Center, Haifa, Israel; 2 Department of Cardiology, Rambam Medical Center, Haifa, Israel

## Introduction

Coronary bypass surgery can be performed less invasively by avoiding cardiopulmonary bypass (off-pump coronary surgery by sternotomy, OPCAB) and by performing a minimally invasive direct coronary artery bypass (MIDCAB) through a left anterior mini-thoracotomy. We compared our early results in patients with single-vessel disease who underwent OPCAB and MIDCAB without cardiopulmonary bypass.

## Materials and Methods

Forty-four patients who underwent left internal mammary artery (LIMA) to left anterior descending (LAD) either by MIDCAB or OPCAB during January 2013–December 2014 were reviewed. Data were obtained retrospectively. Patients undergoing MIDCAB or OPCAB were compared in terms of gender, preoperative risk factors, length of operation, ventilation time, intensive care unit (ICU), and hospital stay, amount of postoperative bleeding, daily postoperative pain (0–10 scale), and troponin and creatinine levels.

## Results

A total of 20 patients in the MIDCAB group and 24 in the OPCAB group were analyzed. There was no mortality in the MIDCAB group, versus two patient deaths in the OPCAB group (*P*= 0.186). No significant differences were observed between the two groups with respect to gender, preoperative risk factors, ventilation time, ICU and hospital stay, amount of postoperative bleeding, or troponin and creatinine levels. The MIDCAB procedure duration was longer compared to the OPCAB [median (IQR): 151 (139.75–176.5) vs. 132 (102–162) min, respectively; *P*=0.029]. Patients undergoing MIDCAB reported greater pain scores on the second postoperative day [5 (2–5) vs. 2 (0–3.5), respectively; *P*=0.027], and similar low pain scores on the fifth postoperative day [0.0 (0–2) vs. 0.0 (0–4), respectively; *P*=0.142].

## Conclusions

MIDCAB procedures can be performed safely in well-selected patients, with low postoperative morbidity and mortality. The higher pain scores during the early postoperative period in the MIDCAB group should be treated more vigorously.

# Abstract: Reconstruction of Complex Oral and Maxillofacial Defects Using PEEK and Titanium Custom-Made Implants

**Published:** 2017-01

**Authors:** Dekel Shilo, Omri Emodi, Ori Blanc, Adi Rachmiel

**Affiliations:** 1 Department of Oral and Maxillofacial Surgery, Rambam Medical Care Center, Haifa, Israel; 2 Ruth & Bruce Rappaport Faculty of Medicine, Technion–Israel Institute of Technology, Haifa, Israel

## Introduction

Reconstructing the craniofacial complex is extremely challenging due to the unique anatomy, presence of vital structures, and the diversity of defects. In craniofacial reconstruction, restoration of aesthetics and function is the primary goal. Auto-grafts are the gold standard for craniofacial skeletal reconstruction. However, auto-grafts have several disadvantages, which led to the research into alloplastic materials. Development of computer-assisted design and computer-assisted manufacturing systems allows for precise preoperative planning and designing of patient-specific implants preoperatively.

## Materials and Methods

Orbital floor and zygoma deficiencies were reconstructed using polyetheretherketone (PEEK) implants. Mandibular defects were reconstructed using titanium implants shaped as a crib, thus allowing addition of artificial and autogenous bone grafts for future dental implant placement.

## Results

Mandibular defects reconstructed using titanium for strength and function exhibited proper mouth opening, function, and aesthetics. In one of the patients, dental implants were designed as part of the patient-specific implant, thus allowing for future implant-supported dental rehabilitation not requiring support of the bone graft. Orbital floor reconstruction resolved the observed late enophthalmos. Zygoma reconstruction successfully eliminated cheek bone deformity.

## Conclusions

Individual computer-assisted design and computer-assisted manufacturing systems for pre-existing facial defects are becoming a popular alternative to auto-grafts. Results are promising and exhibit excellent aesthetic and functional outcomes, while reducing operating time and avoiding donor site morbidity. This procedure provides a simple way to reconstruct complex three-dimensional structures with precision, a result that is difficult to achieve with standard methods.

# Abstract: Solitary Frontal Sinus Fractures Compared to Multiple Facial Fractures, Energy Impact Dependency

**Published:** 2017-01

**Authors:** Efi Weitman, Dekel Shilo, Omri Emodi, Adi Rachmiel

**Affiliations:** 1 Department of Oral and Maxillofacial Surgery, Rambam Medical Center, Haifa, Israel; 2 Ruth & Bruce Rappaport Faculty of Medicine, Technion–Israel Institute of Technology, Haifa, Israel

## Introduction

Frontal sinus fractures account for 2%–15% of maxillofacial injuries, and up to 87% of patients with frontal sinus fractures suffer from associated facial fractures. The majority of classifications used today categorize frontal sinus fractures depending on the integrity of the anterior table, posterior table, and the nasofrontal outflow. We introduce our findings on the relation of frontal sinus fractures with other facial bone fractures correlated with the energy of the trauma. We also suggest a modification to the classifications in use today.

## Materials and Methods

A retrospective study was performed on 24 patients diagnosed with frontal sinus fractures. Treatment for all patients consisted of open reduction and bone fixation. We analyzed population variables, injury etiology, fracture site, associated craniofacial injuries, surgical technique employed, nasofrontal duct handling, and postoperative complications.

## Results

The most frequent etiology was falling accidents, and 58% of fractures involved both the anterior and posterior tables; 66% of patients suffered from associated facial fractures, 50% of frontal sinus fractures were treated by open reduction internal fixation as the only treatment, 33.3% of patients underwent sinus obliteration, and 16.6% were treated with cranialization.

## Conclusions

Frontal sinus fractures resulting from high-energy impact exhibited additional facial bone fractures in 100% of the cases, while fractures following low-energy impact showed involvement of additional facial fractures in only 27% of the cases. In this report, we suggest a modification to the anteroposterior classification of frontal sinus fractures: in addition to the involvement of the anterior and posterior walls and the degree of dislocation, high- and low-energy impact can direct us to the involvement of additional facial fractures and influence the surgical strategy.

# Abstract: Maxillary Ameloblastic Carcinoma Mimicking Periapical Periodontal Lesion

**Published:** 2017-01

**Authors:** Amir Wolff, Yoav Leiser, Omri Emodi, Sharon Akrish, Adi Rachmiel

**Affiliations:** 1 The Department of Oral and Maxillofacial Surgery, Rambam Medical Centre, Haifa, Israel; 2 The Ruth & Bruce Rappaport Faculty of Medicine, Technion–Israel Institute of Technology, Haifa, Israel; 3 The Department of Pathology, Rambam Medical Center, Haifa, Israel

## Introduction

Ameloblastic carcinoma (AC) is a rare primary odontogenic malignancy that combines the histological features of ameloblastoma with cytologic atypia. Maxillary AC is potentially life-threatening by local invasion to vital structures or by distant metastases. We present two cases of maxillary ameloblastic carcinoma that mimicked a periapical odontogenic lesion, and which were misdiagnosed due to their innocent appearance.

## Materials and Methods

An analysis and literature review of all maxillary AC was conducted; we present two additional cases with unusual clinical and radiographic features.

## Results

A PubMed search revealed 45 cases of maxillary AC. Mean age at diagnosis was 55 years, there was a male predilection (ratio 3:1), and the commonest location was posterior maxilla. Radiographically, AC often presented with ill-defined margins, root resorption, and cortical perforation. Recurrence rate was in the range of 23%–38% with a mean time to recurrence of 47.5 months. Our two cases presented radiographically as a periapical radiolucency indistinguishable from an inflammatory lesion, resulting in initial misdiagnosis and delay in treatment. Both cases were treated with maxillectomy with 15 mm safe margins. Follow-up at 48 months revealed no evidence of residual disease.

## Conclusions

Maxillary AC is a relatively rare odontogenic malignancy that presents with diverse clinical and radiographic characteristics. Asymptomatic, long-standing periapical lesions that lack typical signs of acute or sub-acute inflammation may represent a more aggressive entity. In cases such as these, we recommend a low threshold for biopsy in order to improve prognosis and reduce surgical morbidity.

# Abstract: SMART Medicine Produces Evidence for Reduction of Injudicious Use of Diagnostic Tools

**Published:** 2017-01

**Authors:** Ronen Zalts, Shira Buchrits, Dana Ben-Hur, Vera Belsky, Katya Dolnikov, Zaher S. Azzam, Gidon Berger

**Affiliations:** 1 Division of Internal Medicine, Rambam Medical Center, Haifa, Israel; 2 The Ruth & Bruce Rappaport Faculty of Medicine, Technion–Israel Institute of Technology, Haifa, Israel; 3 Intel Electronics, Ltd, Haifa, Israel

## Introduction

The SMART (Specific, Measurable, Agreed, Required, and Timely) Medicine initiative represents a milestone in the development and implementation of a defined method for judicious use of diagnostic tools, aimed to improve the efficiency of medical investigations.

## Objective

To quantify the efficacy of select up-to-the-minute SMART Medicine recommendations.

## Materials and Methods

Retrospective analysis of Rambam Medical Center’s (RMC) proprietary database, concerning the use of diagnostic tools in four topics in the Division of Internal Medicine, between January 2014 to February 2016. The efficacy of SMART Medicine recommendations was examined in the following topics (Figure 1): (1) computed tomography angiography (CTA) for suspicion of pulmonary embolism; (2) liver ultrasound (US) in cases of liver enzyme elevation; (3) the use of C-reactive protein (CRP), hemoglobin A1C (HgBA1C), and thyroid stimulating hormone (TSH) blood tests; (4) the use of various blood tests for the investigation of anemia.

**Figure 1:** Zalts et al. Use of Various Laboratory Tests During the Study Period. The y-axes represent the number of tests corrected per number of admissions. Note that while the analyses were run from January 2014 to February 2016, each test is on a different time line since the recommendations were released at different times.

## Results

The use of diagnostic tools in the four examined topics has declined; the reduction in the number of tests directly translates to a monthly saving of $7,500. Use of CTA for suspicion of pulmonary embolism decreased from 34 to 26 examinations a month (*P*=0.02). An average monthly decrease of 8 (15%) in liver US examinations in cases of liver enzyme elevation was found (*P*=0.03), while the number of CRP, HgBA1C, and TSH tests conducted were significantly lower during the study period (P<0.001) compared to the time period before our recommendations were released. The investigation of anemia has become more focused, with a significant decline (20%) in the number of folic acid, vitamin B12, and ferritin tests. The decline in usage of various laboratory tests are shown in Figure 1 (next page).

## Conclusions

The SMART Medicine campaign has led to improved efficiency of medical investigations in these four topics. These results are encouraging, with regard to extending the SMART Medicine campaign to other topics, widening the implementation of the recommendations to more divisions at RMC, and consideration of introducing SMART Medicine methodology to medical schools’ syllabi.
